# Identification of Homeobox Genes Associated with Lignification and Their Expression Patterns in Bamboo Shoots

**DOI:** 10.3390/biom9120862

**Published:** 2019-12-11

**Authors:** Xiurong Xu, Yongfeng Lou, Kebin Yang, Xuemeng Shan, Chenglei Zhu, Zhimin Gao

**Affiliations:** 1 Institute of Gene Science and Industrialization for Bamboo and Rattan Resources, International Center for Bamboo and Rattan, Beijing 100102, China; xuxiurong@icbr.ac.cn (X.X.); yangkebin@icbr.ac.cn (K.Y.); shanxuemeng@icbr.ac.cn (X.S.); zhuchenglei@icbr.ac.cn (C.Z.); 2 State Forestry and Grassland Administration / Beijing Key Open Laboratory on the Science and Technology of Bamboo and Rattan, Beijing 100102, China; 3 Jiangxi Academy of Forestry, Nanchang 330013, China; louyf1983@163.com

**Keywords:** *Phyllostachys edulis*, homeobox gene, lignification, expression analysis

## Abstract

Homeobox (HB) genes play critical roles in regulating various aspects of plant growth and development. However, little is known about HB genes in bamboo. In this study, a total of 115 HB genes (*PeHB001*–*PeHB115*) were identified from moso bamboo (*Phyllostachys edulis*) and grouped into 13 distinct classes (BEL, DDT, HD-ZIP I–IV, KNOX, NDX, PHD, PINTOX, PLINC, SAWADEE, and WOX) based on the conserved domains and phylogenetic analysis. The number of members in the different classes ranged from 2 to 24, and they usually varied in terms of exon–intron distribution pattern and length. There were 20 conserved motifs found in 115 PeHBs, with motif 1 being the most common. Gene ontology (GO) analysis showed that *PeHB*s had diverse molecular functions, with 19 *PeHB*s being annotated as having xylem development, xylem, and phloem pattern formation functions. Co-expression network analysis showed that 10 of the 19 *PeHB*s had co-expression correlations, and three members of the KNOX class were hub proteins that interacted with other transcription factors (TFs) such as MYB, bHLH, and OVATE, which were associated with lignin synthesis. Yeast two-hybridization results further proved that PeHB037 (BEL class) interacted with PeHB057 (KNOX class). Transcriptome expression profiling indicated that all *PeHB*s except *PeHB017* were expressed in at least one of the seven tissues of moso bamboo, and 90 *PeHB*s were expressed in all the tissues. The qRT-PCR results of the 19 *PeHB*s showed that most of them were upregulated in shoots as the height increased. Moreover, a KNOX binding site was found in the promoters of the key genes involved in lignin synthesis such as *Pe4CL*, *PeC3H*, *PeCCR*, and *PeCOMT*, which had positive expression correlations with five KNOX genes. Similar results were found in winter bamboo shoots with prolonged storage time, which was consistent with the degree of lignification. These results provide basic data on *PeHB*s in moso bamboo, which will be helpful for future functional research on *PeHB*s with positive regulatory roles in the process of lignification.

## 1. Introduction

Homeobox (HB) genes are widely found in almost all eukaryotes, and have been divided into 11 gene classes in animals [1]. Initially, the HB gene was obtained through the hybridization of a somatic mutant and homologous mutant of the fruit fly, which was involved in the regulation of the position, shape, and number of animal somatic segments [2,3]. Then, homologs of HB genes were also isolated from evolutionarily distant species like plants and fungi [4,5]. There are 180 bp in the HB gene encoding a conserved DNA-binding domain of 60 aa known as a homeodomain (HD), which characterizes a large family of transcription factors (TFs). The characteristic three-dimensional structure of HD contains three alpha helices, of which the second and third form a helix-turn-helix motif [6,7]. Many HB genes in plants encode TFs containing HD that can bind to *cis*-regulated regions of their target genes, which play regulatory roles in various biological processes of plant growth and development [8]. In the evolutionary process of plants, HB genes have been differentiated into 14 distinct classes with various structures characterized by conserved intron–exon structure and a unique domain architecture, including BEL, DDT, HD-ZIP I–IV, KNOX, LD, NDX, PHD, PINTOX, PLINC, SAWADEE, and WOX [9]. 

The HD-ZIP classes are characterized by a leucine-zipper conserved domain adjacent to the C-terminus of the HD, while the other 10 classes not only contain the HD, but also have their own specific conserved domains. For example, PLINC is distinguished from other zinc finger classes by the substitution of the usually conserved homeodomain residue F49, with a methionine residue and the insertion of one amino acid between helix 1 and helix 2 [9]. The members of HD-ZIP classes have been shown to play important roles in regulating plant growth, development, and environmental responses [10,11,12,13,14,15,16,17,18]. The members of the WOX class have a derived function of stem cell control besides embryonic patterning, stem cell maintenance, and organ formation, indicating that gene amplification followed by functional diversification is a major force in their evolution [19,20]. The TALE (three–amino acid loop extension) class (BEL and KNOX) is characterized by three extra residues between helix 1 and helix 2 [6,21], among which KNOX plays important roles in plant cell growth and development, including cell wall formation and lignification [22,23]. Moreover, studies in *Arabidopsis* and the peach have shown that AtBP and PpKNOPE1 can interact with the typical KNOX DNA-binding site (TGACAGC) in *4CL*, *COMT*, and *LAC* to regulate their expression during lignification [24,25].

Moso bamboo (*Phyllostachys edulis*) belongs to the subfamily Bambusoideae of the *Poaceae* family, with characteristics of fast growth and excellent materiality, it can grow about 20 m in 1.5 months [26] and is a good alternative to wood. With the publishing of the moso bamboo genome [27] and the bamboo genome database (BambooGDB) [28], the work on gene identification at the whole-genome level is being greatly accelerated. Genome-wide analysis of IQD [29], SBP-like [30], TCP [31], heat shock [32], and MYB [33] has been performed in moso bamboo. Although identification and function analysis of HB genes have been widely carried out in many plant species such as rice, grapes, and carrots [34,35,36], a comprehensive understanding of the status of HB genes is lacking in moso bamboo, and those associated with lignification are still unclear. To understand the HB genes in moso bamboo, comprehensive analyses of HB genes including the molecular characteristics, gene structure, conserved domain, evolutionary relationship, and expression profile were conducted in this paper. The expression of HB genes and their target genes related to lignin synthesis were further analyzed in moso bamboo shoots undergoing lignification, which will be helpful for further study of the molecular mechanism of HB genes involved in bamboo lignification.

## 2. Materials and Methods

### 2.1. Identification of HB Genes in Moso Bamboo

To identify the members of HB genes in moso bamboo, the previously identified HB genes of *Arabidopsis thaliana* and *Oryza sativa* were retrieved from the database TAIR 10.0 (The *Arabidopsis* Information Resource, https://www.arabidopsis.org/) and RGAP 7.0 (Rice Genome Annotation Project, http://rice.plantbiology.msu.edu/), respectively [37,38], and were used as queries in BLAST searches against the database of the moso bamboo genome (BambooGDB, http://bamboo.bamboogdb.org/) [28]. The sequences were selected for further analysis if the E-value was less than 1 × 10^−10^. Then, all putative sequences were validated by the Pfam program (http://pfam.xfam.org/) [39] and aligned using Clustal X [40] to remove redundant sequences. In addition, the open reading frame (ORF), molecular weight (MW), and isoelectric point (pI) parameters of each HB protein were calculated by the online program ExPasy (http://www.expasy.org/tools/) [41].

### 2.2. Sequence Alignment, Phylogenetic Analysis, Gene Structure, and Conserved Motif Prediction

To explore the conservation of obtained sequences, multiple alignments were carried out using the full-length translated protein sequences of HB proteins from moso bamboo, *Arabidopsis*, and rice by Clustal X 2.1 [40]. Phylogenetic trees were constructed using MEGA 7.0 software (https://www.megasoftware.net/) with the Maximum-likelihood (ML) method and the bootstrap test was replicated 1000 times [42]. The Gene Structure Display Server (GSDS, http://gsds.cbi.pku.edu.cn) was used to analyze the exon/intron of HB genes obtained from moso bamboo [29]. The conserved motifs of moso bamboo HBs were predicted using the MEME (Multiple Em for Motif Elicitation) system (http://meme-suite.org/tools/meme) with the following parameter settings, which included the distribution of motifs: zero and one per sequence; maximum number of motifs to find: 40; minimum width of motif: 6; and maximum width of motif: 120 [43]. 

### 2.3. GO Analysis, Co-Expression Network and Target Gene Prediction

The proteins encoded by the HB genes of moso bamboo were annotated using the PLAZA 4.0 to assign GO terms (https://bioinformatics.psb.ugent.be/plaza/) with the software-set parameters [44]. The BambooNET (http://bioinformatics.cau.edu.cn/bamboo/cytoscape/network.php) web site [45] and Cytoscape software (https://cytoscape.org/) [46] were used for the co-expression network analysis of moso bamboo HB genes. The promoter sequences of key genes (*PAL*, *4CL*, *C3H*, *C4H*, *HCT*, *CCR*, *CCoAOMT*, *CAD*, *F5H*, *COMT*, and *LAC*) related to lignin biosynthesis in moso bamboo were downloaded from PLAZA 4.0 [44], and the specific DNA-binding site of HB TFs was searched for in the promoters using the PlantCARE database (http://bioinformatics.psb.ugent.be/webtools/plantcare/html/) [47].

### 2.4. Tissue Specific Expression Analysis Based on Transcriptome Data

To investigate the expression of HB genes in the different tissues and developmental stages of moso bamboo, the transcriptome data generated from leaves, early panicles, advanced panicles, roots, rhizomes, 20-cm shoots and 50-cm shoots were downloaded from the NCBI Short Read Archive (SRA) [27] and used for further gene expression analysis. The gene expression abundance was calculated by the Reads Per Kilobase per Million mapped reads (RPKM) value of each HB genes. For the convenience of running the statistics, logarithm (Log) was used for each expression as base 2. The heatmap of HB genes was exhibited using Matrix2png (https://matrix2png.msl.ubc.ca/bin/matrix2png.cgi) [48].

### 2.5. Sample Collection

To examine the expression levels of different HB genes associated with lignin biosynthesis in moso bamboo shoots during the lignification process, the basal parts of shoots with different heights (0.2, 1.0, 3.0, and 6.7 m) were collected from the bamboo forest experimental site of Jiangxi Academy of Forestry located in Nanchang, Jiangxi Province, China. In addition, winter bamboo shoots (0.3 m in length, 0.1 m in diameter) without any blemishes and disease were harvested and stored in darkness in perforated plastic bags at 25 °C. The basal parts of the first internode of shoots were collected as samples after 0, 3, 6, and 12 d for RNA isolation and lignin content determination. Each sample was three technical replicates with five shoots per replicate, frozen immediately in liquid nitrogen and stored at –80 °C. Meanwhile, formalin–acetic acid–alcohol (FAA) was used to fix the samples collected from the same part, which were stored at 4 °C for further morphology analysis.

### 2.6. RNA Isolation and qRT-PCR Analysis

The total RNA was extracted using the plant total RNA extraction kit (TaKaRa, Tokyo, Japan) according to the manufacturer’s instructions. The integrity of the total RNA was analyzed on a 1.0 % agarose gel. RNA quantity was determined using a NanoDrop 2000 Spectrophotometer (Thermo Scientific, Waltham, MA, USA). Total RNA was used for the synthesis of cDNA by a reverse transcription system (Promega, Madison, WI, USA). 

Quantitative real-time PCR (qRT–PCR) analysis was performed using the *PeNTB* gene as an internal control [49]. The specific primer sequences of HB genes are listed in Table S1. A qTOWER2.2 system (Analytik, Jena, Germany) was used for all qRT-PCR assays. Each 10 μL reaction volume included 5.0 μL of 2× SYBR Green 1 Master Mix, 0.8 μL of cDNA, 0.2 μL of forward and reverse primer (10.0 mM), and 4.0 μL of ddH_2_O. PCR was performed with cycling parameters as follows: 95 °C for 10 min, followed by 40 cycles at 95 °C for 10 s and 60 °C for 10 s. The expression levels were calculated using the 2^−ΔΔCT^ method [50]. Each PCR assay was run in triplicate for three independent biological repeats.

### 2.7. Measurement of Lignin Content and Histological Observation

The winter bamboo samples were dried in an oven at 80 °C, until there was no change in their weight. The samples were ground into a powder with a mortar and pestle, then filtered with a 40-mesh sieve and used for further lignin content measurement using the acetyl bromide method [28]. The data were presented as means of three biological replicates.

The fixed samples were taken out and rinsed with sterile water 3–5 times to remove the FAA fixative on the surface. Then we cut the samples into a pyramid shape and fixed them on the tray with glue. The samples were sectioned using a vibratome (Leica VT1000S, Nußloch, Germany) at a thickness of 20 μm. The sections were stained with 0.01% (*w*/*v*) Toluidine Blue O [51], and digital images were captured using an Olympus CX31 microscope.

### 2.8. Yeast Two-Hybridization

Based on the analysis of co-expression network, the hub HB predicted with protein–protein interaction (PPI) were selected for further validation by using yeast two-hybridization. To explore the transcriptional activity of the genes, the yeast expression vectors were constructed in pGBKT7 vectors. Primer sequences for the construction of expression vectors were designed as listed in Table S2. The constructed vectors, positive control of pGBKT7-53 and empty pGBKT7 vectors were independently transformed into the yeast strain AH109, which was inoculated onto SD/−Trp and SD/−Trp/−His/−Ade/X-α-GAL media for the analysis of transcriptional activity, respectively. The genes without autonomous transcriptional activities were constructed using pGBKT7 or pGADT7, respectively. The recombinant plasmids were co-transformed into yeast strain AH109 and then plated on selective media (SD/−Leu/−Trp and SD/−Leu/−Trp/−His/−Ade/X-α-GAL) to screen for positive clones [52].

### 2.9. Statistical Analysis

IBM SPSS Statistics (Version 21.0, IBM, Armonk, NY, USA) was used for statistical analysis, and the mean and standard deviations of three biological replicates are presented. Significant differences are indicated at * *p* < 0.05 and ** *p* < 0.01.

## 3. Results

### 3.1. Identification of HB Genes in Moso Bamboo

After a series of comprehensive comparison analyses, including the BLAST search, conserved domain analysis, sequence multiple alignment and phylogenetic analysis, a total of 115 HB genes were identified in moso bamboo, which were named *PeHB001* to *PeHB115* according to their physical locations on the genome scaffolds. The proteins encoded by *PeHB*s ranged from 197 aa to 542 aa in length, with an average of 357.5 aa. The basic characteristics of PeHBs including isoelectric point (pI), molecular weight (MW), instability index, grand average of hydropathy (GRAVY) value, and subcellular localization are shown in Table S3. In addition, the prediction of subcellular localization showed that 112 (97%) of the 115 PeHBs were located in the nucleus, while PeHB043, PeHB060, and PeHB080 were located in the mitochondria, extracellular, and plasma membrane, respectively (Table S3).

### 3.2. Multiple Sequence Alignment and Phylogenetic Analysis of PeHBs

To investigate and identify the characteristics of homologous domains in PeHBs, the multiple sequence alignment was carried out. The result demonstrated that the specific domains of 13 identified classes (BEL, DDT, HD-ZIP I–IV, KNOX, NDX, PHD, PINTOX, PLINC, SAWADEE, and WOX) were found in the sequences of PeHBs, and the representative homeodomain sequences of moso bamboo, *Arabidopsis*, and rice are shown in Figure S1.

**Figure 1:**
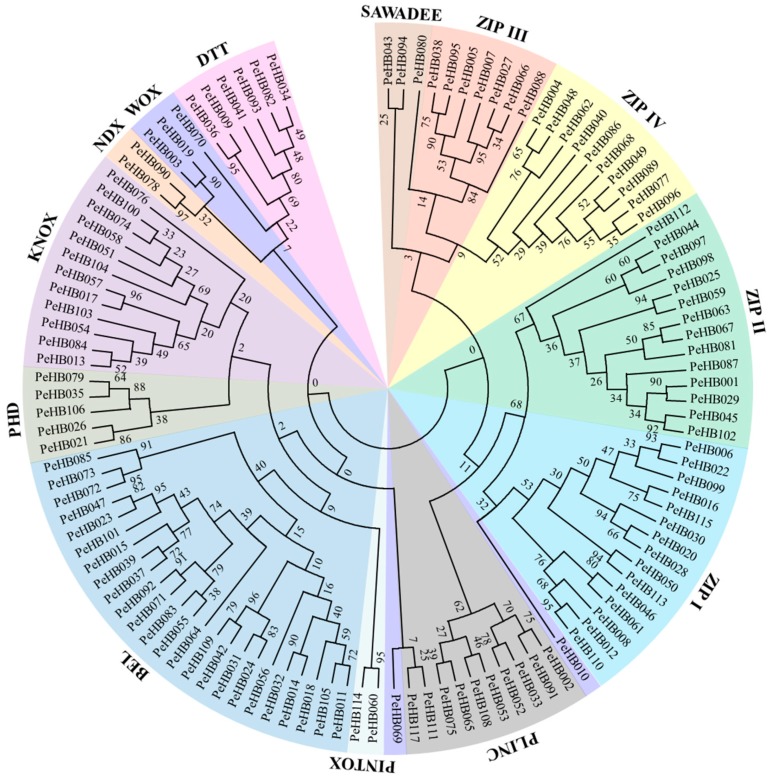
Phylogenetic tree based on the amino acid sequences of PeHB proteins. Geometric figures of different classes were colored to represent different homeobox classes. The phylogenetic tree was constructed via MEGA 7.0 on the basis of the amino acid sequences of PeHBs with the maximum likelihood (ML) method. Bootstrap analysis was conducted with 1000 replicates.

The phylogenetic analysis showed that all 329 HB amino acid sequences from moso bamboo (115), *Arabidopsis* (107), and rice (107) were classified into 14 classes based on the similarity of their HD domain and the presence of specific domains (Figure S2). Most members of the main classes were represented by bamboo, *Arabidopsis*, and rice, suggesting that each class had a common ancestor before the divergence of these plant lineages. The 115 PeHBs were distributed in 13 classes, of which four (HD-ZIP I–IV) were grouped into the HD-ZIP class and two (KNOX and BEL) formed the TALE class (Figure 1). The members of PeHBs in most other classes (BEL, DDT, HD-ZIP I–IV, KNOX, PHD, PLINC, and SAWADEE) were similar to those in *Arabidopsis* and rice (Table S4). However, the exceptions were found in the WOX class, which had 16 members of *Arabidopsis*, 19 members of rice, and only five members of moso bamboo. Moreover, the member of the LD class was not found in moso bamboo.

### 3.3. Gene Structure and Conserved Motif of PeHB Genes

The analysis of gene structure showed that there was a significant diversity among the members of *PeHB*s (Figure S3). Twenty different exon–intron distribution patterns were found, and the intron number of *PeHB*s varied from 0 to 36. Most members of the PLINC, HD-ZIP I, and HD-ZIP II classes had fewer introns and a shorter gene length, while those in the DDT and HD-ZIP III classes had more introns and a longer gene length. The most complex gene structure was *PeHB078* in the NDX class, which was 22,071 bp and contained 36 introns. Most members of the same class had a similar gene structure, but there were a few exceptions. For example, of the three members of the SAWADEE class, *PeHB094* was 1429 bp and contained only one intron, while *PeHB043* and *PeHB080* were 3651 and 16,491 bp with eight and 31 introns, respectively, indicating that they were relatively more diverse.

**Figure 2:**
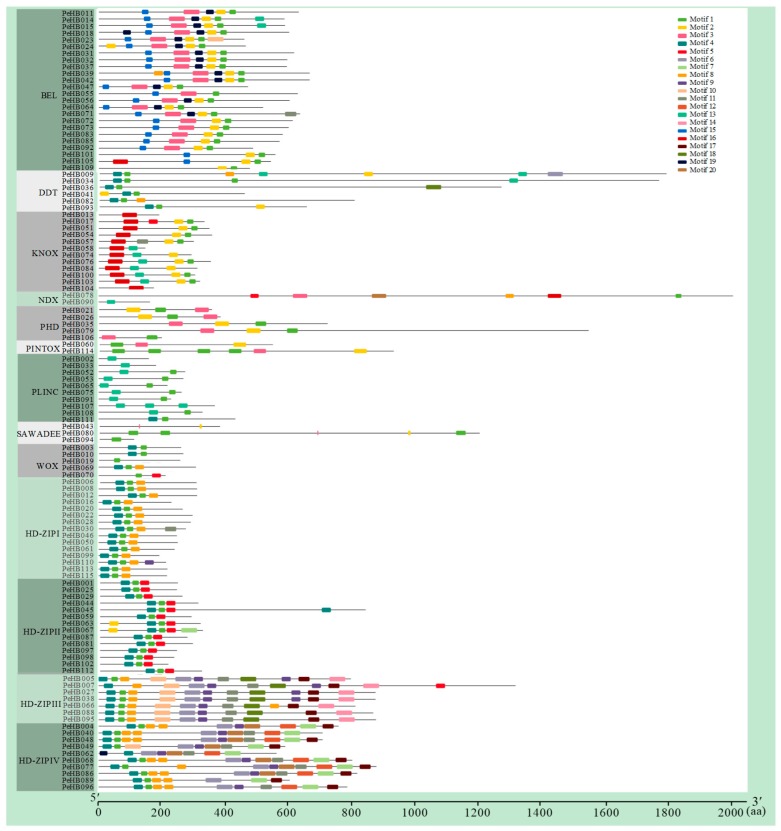
Motifs were identified using MEME software and distinguished by different colors.

In addition, the conserved motifs in the proteins encoded by *PeHB*s were analyzed, and a total of 20 conserved motifs were found (Figure 2). Further analysis indicated that the number of amino acid residues in each conserved motif was different; the shortest one was 15 aa in motif 2, and the longest was 50 aa in eight motifs (3, 6, 7, 10, 12, 14, 18, and 20). Among the 20 motifs, motif 1 (GLTPRQVSNWFQNRRARLKKK) with highly conservative amino acids (W10 and F11) was part of the HD conserved domain, which was found in 95 PeHBs, accounting for 83% of the total. Motifs 13, 15, and 16 were specific to ZIP III, BEL, and KNOX, respectively (Figure S4).

### 3.4. Expression Profiles of PeHBs in Different Tissues

**Figure 3:**
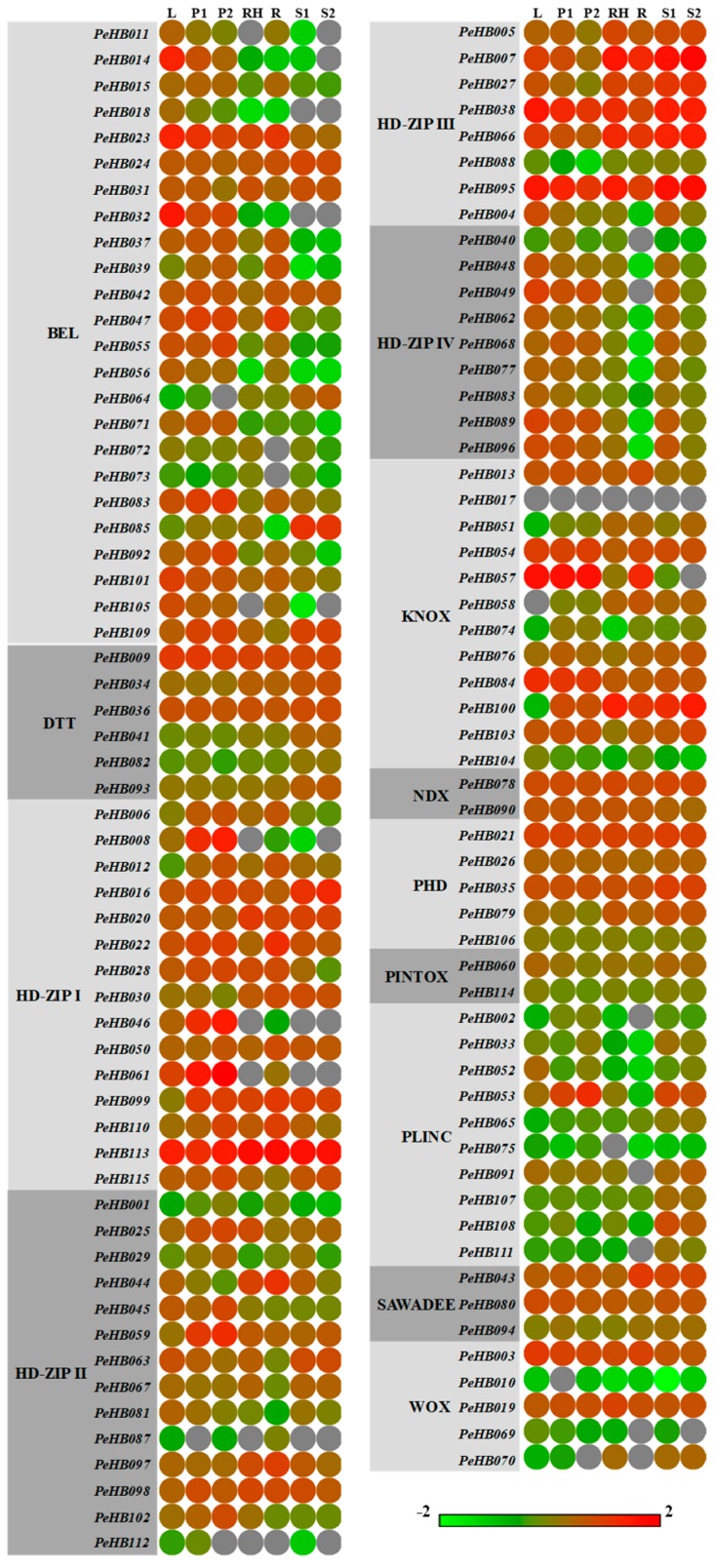
Tissue-specific expression analysis of *PeHB*s in moso bamboo. Heatmap represented for the expression of 115 *PeHB*s in different tissues. The color scale at the bottom of the figure represents log_2_ expression values, with the color from blue to red indicating a low to high level of transcript abundance. L: leaves; P1: early panicles; P2: advanced panicles; R: roots; RH: rhizomes; S1: 20-cm shoots; S2: 50-cm shoots.

To compare the expression levels of *PeHB*s in different tissues of moso bamboo, the publicly available transcriptome data were used for further analysis. The result showed that the gene expression levels of *PeHB*s were diverse in different tissues (Figure 3). All the *PeHB*s except for *PeHB017* were expressed in at least one of seven tissues; *PeHB001*, *PeHB005*, *PeHB008*, *PeHB010*, and *PeHB012* were not detected in the leaves, panicles, rhizomes, roots, and bamboo shoots, respectively. Furthermore, 90 genes were expressed in all tissues, but their expression levels varied greatly among tissues, and most *PeHB*s displayed a tissue-specific expression pattern. For example, most members of the BEL class were highly expressed in the leaves and panicles, and relatively low in the other four tissues, especially in bamboo shoots.

*PeHB*s belonging to the PLINC class showed dominant expression in the bamboo shoots, and were relatively low in the other tissues. In addition, some *PeHB*s, such as the members of the HD-ZIP III, SAWADEE, and NDX classes, were highly expressed in all tissues. However, the expression patterns of members of the same class were different among the tissues. For instance, in the KNOX class, *PeHB054*, *PeHB057*, and *PeHB084* were highly expressed in the leaves, while *PeHB051*, *PeHB058*, *PeHB074*, and *PeHB100* had low expression in the leaves. Interestingly, the expression levels of the members of the PHD and HD-ZIP IV classes in the roots were significantly lower than those in other tissues, and *PeHB040*, *PeHB049*, *PeHB072*, and *PeHB073* were not detected in the roots.

### 3.5. Function Prediction of PeHBs Based on GO Analysis

Gene Ontology (GO) enrichment analysis was performed to characterize the main biological functions of *PeHB*s. The information on the GO annotation of the *PeHB*s originated from the PLAZA was used for further analysis. Among the 115 *PeHB*s, the GO terms of 113 genes were found (Table S5). More than 65% of *PeHB*s were found to be enriched in the GO category of the biological process (BP) (Figure S5). The results suggested that *PeHB*s were involved in the biological process, molecular function, and cellular component (Table S6). Moreover, ‘sequence-specific DNA binding’ (GO:0043565), ‘transcription factor activity, sequence-specific DNA binding’ (GO:0003700), and ‘protein binding’ (GO:0005515) were the most common terms found in molecular function, suggesting that these *PeHB*s as TFs have strong transcriptional activity and can bind to specific DNA to perform functions. Among all the molecular functions, the number of genes regulating ‘xylem development’ (GO:0010089) and ‘xylem and phloem pattern formation’ (GO:0010051) is 19; these were then selected for co-expression analysis and expression pattern validation in shoots of the moso bamboo. 

### 3.6. Co-Expression and PPI Networks of PeHBs

**Figure 4:**
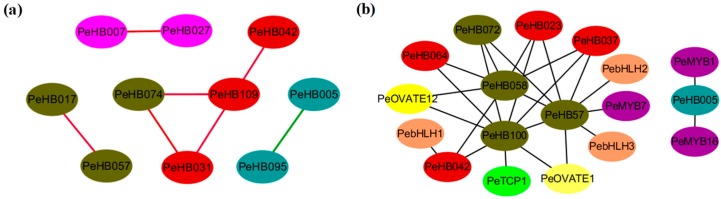
Predicted network of 19 *PeHB*s. (**a**) A co-expression network of 19 *PeHB*s. Green, red, pink and turquoise shading represent the KNOX, BEL, and ZIP classes, respectively. The red and green lines represent positive and negative regulation, respectively. (**b**) A protein–protein interaction (PPI) network of 19 PeHBs and other transcription factors (TFs). Shading with different colors represents different TFs.

To construct the gene networks involved in lignification, the 19 *PeHB*s annotated as GO:0010089 and GO:0010051 were selected. The co-expression network diagrams were generated basing on the transcriptome data [45] (Figure 4a, Table S7). The result showed that 10 of the 19 *PeHB*s had co-expression correlations. Six gene pairs were found to have positive co-expression with each other, including *PeHB07* and *PeHB027*, *PeHB017* and *PeHB057*, *PeHB031* and *PeHB109*, *PeHB042* and *PeHB109*, *PeHB074* and *PeHB031*, and *PeHB074* and *PeHB109*. The only negative co-expression gene pair was *PeHB005* and *PeHB095*. These results indicated that the co-expressed *PeHB*s might work together in regulating lignification in the cell wall of moso bamboo.

To further investigate the interaction of 19 PeHBs with other proteins in moso bamboo, a PPI network was constructed. The result showed that there were 18 nodes and 26 edges. The nodes represented the PeHBs and other TFs, the edge was the interaction between two proteins (Figure 4b, Table S8). Based on the connection between nodes, 18 nodes can be distinctly classified into two groups. In one group, 15 of 18 nodes were connected, which contained eight PeHBs including four members of the KNOX class and four members of the BEL class. Three members of the KNOX class were the hub proteins that interacted with the other PeHBs and TFs such as MYB, bHLH, OVATE etc. Another group only contained three nodes and two edges, in which PeHB005 interacted with both PeMYB1 and PeMYB16. The homologous genes of MYB, bHLH, and OVATE in rice have been confirmed to be associated with lignin synthesis [53,54,55], suggesting that the regulation of lignin synthesis in moso bamboo may be a complicated process associated with various TFs besides PeHBs.

### 3.7. PeHB037 Interacted with PeHB057 as Co-Transcription Factors

Four members (PeHB057, PeHB058, PeHB072, and PeHB100) of the KNOX class presented in the PPI network were selected, they were cloned and sequenced to confirm their complete ORF, and then constructed in pGBKT7. The AH109 yeast cells containing the recombinant plasmids were tested on the selective media. The results showed that the positive control group (pGBKT7-53) exhibited visible blue colonies with satisfactory growth states on the SD/−Trp/−His/−Leu/X-α-GAL medium, while the transformants of pGBKT7::KNOXs and pGBKT7 were unable to survive (Figure S6), indicating that four members of the KNOX class did not possess autonomous transcriptional activity in yeast. 

**Figure 5:**
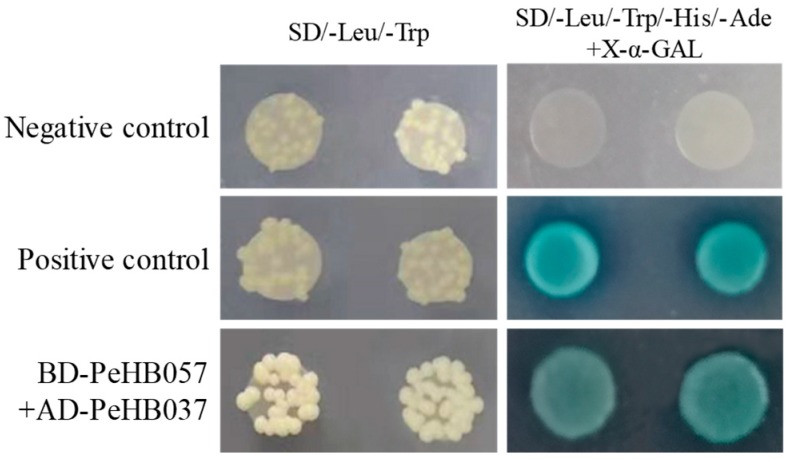
The interaction between PeHB037 and PeHB057 in yeast.

To investigate the PPI between different PeHBs, the predicted PPI pair of PeHB037 (BEL class) and PeHB057 (KNOX class) was used for further validation by yeast two-hybrid. The ORFs of PeHB037 and PeHB057 were constructed into activation domain and DNA-binding domain plasmid vectors, respectively. As depicted in Figure 5, AD-PeHB037 and BD-PeHB057 co-transformed AH109 yeast cells grew well and turned blue on a SD/−Leu/−Trp/−His/−Ade/X-α-GAL selective medium, similar to the positive control, suggesting that PeHB037 interacted with PeHB057 in yeast.

### 3.8. PeHBs Associated with Lignin Biosynthesis Expressed in Shoots of Moso Bamboo

**Figure 6:**
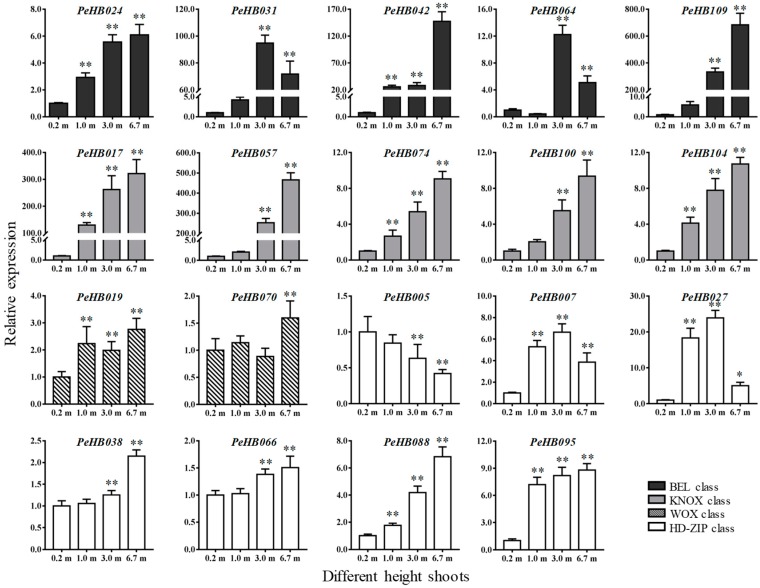
Expression analysis of *PeHB*s in shoots with different heights. Average and error bars represent the standard deviation of three biological replicates. The y-axis and x-axis indicates relative expression levels and the different heights of the base shoots, respectively (* *p* < 0.05, ** *p* < 0.01).

According to GO analysis, the expression of 19 *PeHB*s related to the biosynthesis and deposition of the secondary cell wall were selected for further validation using qRT-PCR, with *PeNTB* as the reference gene. The results demonstrated that the expression levels of most *PeHB*s increased significantly along with the shoot growth, and reached the highest expression level in 6.7-m shoots (Figure 6). Compared to the 0.2-m shoots, all expression levels of the members of the BEL and KNOX classes were upregulated in the 6.7-m shoots. In particular, *PeHB042*, *PeHB109*, *PeHB017* and *PeHB057* were markedly upregulated by more than 147-, 683-, 321-, and 465-fold compared to the expression in 0.2-m shoots, respectively. In addition, the expression levels of *PeHB031* and *PeHB064* increased gradually and reached the maximum in 3.0-m shoots, followed by a decline. *PeHB019* and *PeHB070* of the WOX class showed a similar trend with an increase first, followed by a decrease, finally reaching the maximum in 6.7-m shoots. All the members of the ZIP class except *PeHB005* were upregulated, among which *PeHB038*, *PeHB066*, *PeHB088*, *and PeHB095* showed a rising trend, while *PeHB007* and *PeHB027* were significantly upregulated and reached the maximum in 3.0-m shoots, and then decreased in 6.7-m shoots with obviously higher levels than those in 0.2-m shoots.

**Figure 7:**
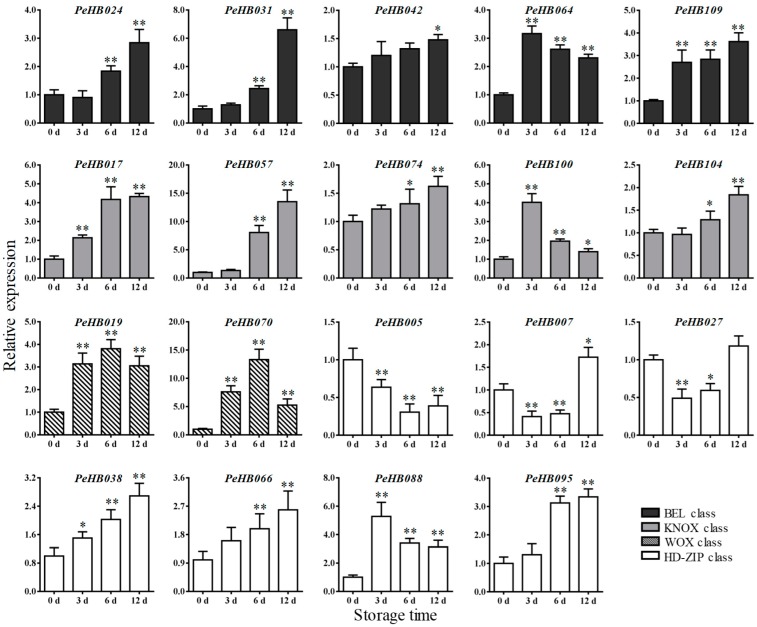
Expression analysis of *PeHB*s in winter bamboo shoots during storage. Average and error bars represent the standard deviation of three biological replicates. The y-axis and x-axis indicate the relative expression levels and the storage days, respectively (* *p* < 0.05, ** *p* < 0.01).

To further understand whether *PeHB*s were involved in the lignification, we also investigated the expression patterns of the 19 *PeHB*s in winter bamboo shoots during storage at 25 °C. As shown in Figure 7, the expression levels of all the *PeHB*s in winter shoots had obviously changed during storage and were upregulated at the end of storage–except *PeHB005*, which showed a downregulation. A total of nine genes (*PeHB017*, *PeHB031*, *PeHB038*, *PeHB042*, *PeHB057*, *PeHB066*, *PeHB074*, *PeHB095*, and *PeHB109*) showed an increasing expression trend along with the storage time, and reached the highest level at the end of the storage (12 d). For example, *PeHB031*, *PeHB057*, and *PeHB095* were upregulated more than 6.5-, 14.8-, and 3.2-fold in winter shoots at 12 d compared to those at 0 d, respectively. The expression levels of *PeHB064*, *PeHB088*, and *PeHB100* upregulated rapidly and reached the maximum at 3 d, followed by a decline; those of *PeHB019* and *PeHB070* of the WOX class showed a similar trend of upregulation, with the maximum at 6 d, then a decline at 12 d. The expression of five genes in winter shoots during storage showed a trend of first decreasing and then rising, in which *PeHB024* and *PeHB104* were first slightly downregulated and then significantly upregulated to reach the maximum at the end of the storage (12 d); *PeHB007* and *PeHB027* were first significantly downregulated and then upregulated; *PeHB005* was the only one that was significantly downregulated. *PeHB024* and *PeHB104* were downregulated slightly at 3 d, and then increased significantly. 

### 3.9. Lignification in Winter Bamboo Shoots during Storage

Bamboo shoots are the immature culm of the bamboo; the process of lignification occurs with the increase in height and the storage days. The lignification process was mainly characterized by the deposition of lignin in the cell wall, which has been proved in different height shoots [33]. Therefore, the collected winter shoots stored at 25 °C were used to analyze the lignin changes after 0 d, 3 d, 6 d, and 12 d.

**Figure 8:**
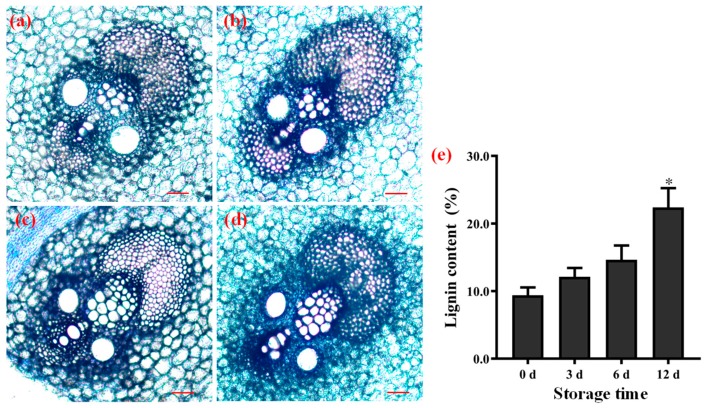
Analysis of the lignification in winter bamboo shoots during storage. (**a**–**d**) The transverse sections of vascular bundle in the stored shoots after 0 d, 3 d, 6 d, and 12 d. Scale bar: 100 μm. (**e**) The lignin content. Asterisks indicated a significant difference between the storage shoots and the fresh shoots (* *p* < 0.05).

The histochemical staining results showed that the stained area and depth of vascular bundles in winter shoots gradually increased along with the storage days (Figure 8a–d). Meanwhile, the content of lignin in winter shoots also increased with the prolonged storage days. The lignin content at 12 days of storage is 22.38%, which is significantly higher than in fresh shoots (9.38%) (* *p* < 0.05) (Figure 8e). The increasing lignin was consistent with the upregulated expression of most *PeHB*s (14 of 19 *PeHB*s) in winter shoots (Figure 7). These results indicated that the winter bamboo shoots had undergone lignification during storage, in which the *PeHB*s might be involved in the regulating process. Moreover, the content of cellulose was increased and that of hemicellulose gradually decreased along with the storage days (Figure S7), which was similar to the reported results [56].

### 3.10. Structural Genes Related to Lignin Synthesis Expressed in Bamboo Shoots during Lignification

**Figure 9:**
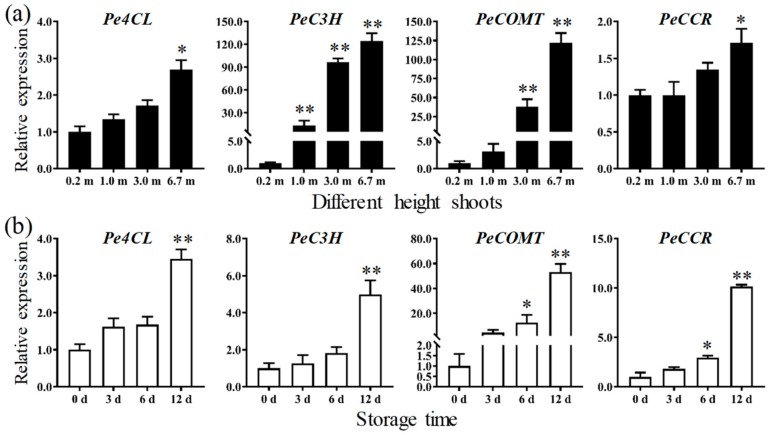
Expression analysis of the genes related to lignin synthesis in bamboo shoots (* *p* < 0.05, ** *p* < 0.01). (**a**) Different height bamboo shoots. The y-axis and x-axis indicate relative expression levels and the different heights of the base shoots, respectively. (**b**) Winter bamboo shoots during storage. The y-axis and x-axis indicate relative expression levels and the storage days, respectively.

Based on the analysis of regulatory elements in the promoter sequences of key genes related to lignin biosynthesis, only the DNA binding motif of KNOX (TGACAGC) was found in the promoters of four lignin synthesis genes, *Pe4CL* (PH01000809G0020), *PeC3H* (PH01001724G0360), *PeCCR* (PH01001334G0240), and *PeCOMT* (PH01001283G0360), indicating that they might be regulated by KNOX. The qRT-PCR results showed that the expression of *Pe4CL*, *PeC3H*, *PeCCR*, and *PeCOMT* increased obviously with the process of lignification. In particular, the expression of *PeCOMT* in 6.7 m shoots was markedly upregulated to more than 53-fold of that in 0.2 m shoots, and that in winter shoots after storage for 12 days was 122-fold of that in the fresh shoots (Figure 9). These results indicated that the four lignin synthesis genes were positively correlated with the five *PeHB*s of the KNOX class in the increasing bamboo shoots (Figure 6) and three *PeHB*s (*PeHB017*, *PeHB057*, and *PeHB074*) in winter shoots (Figure 7), which provides a powerful evidence for the *PeHB*s being involved in the regulation of the lignin process.

## 4. Discussion

The HB genes are common in plants, and play important roles in regulating cell differentiation, embryonic shoot meristem formation, embryo patterning, vascular development, floral organogenesis, and fruit ripening [18,57]. Therefore, fully understanding the situation of HB genes in different species, and making clear the specific characteristics of each HB gene, will help us to explore the specific gene function in plants. Up to now, HB genes have been widely studied in *Arabidopsis* [58] and rice [59], but only the ZIP and WOX classes have been studied in moso bamboo [60,61], so it is necessary to identify all the HB members at a whole-genome level in moso bamboo.

### 4.1. Diverse Features of HB Genes in Moso Bamboo

We first identified 115 HB genes from the genome of moso bamboo, which was more than the number of members of *Arabidopsis*, and rice (107 each) [34,62]. The number of members of the HD-ZIP class was the largest in moso bamboo, *Arabidopsis*, and rice. No members belonging to the LD class were found in moso bamboo, suggesting that gene deletions may have occurred during its evolution. The members of the BEL, PHD, and SAWADEE classes of moso bamboo were all more numerous than in those classes of *Arabidopsis* and rice. In particular, the members of the BEL class were almost twice as numerous of those in *Arabidopsis* and rice, which indicated that gene expansion occurred in the BEL class of moso bamboo, maybe leading to the functional redundancy. The members of the WOX class in most seed plants had been divided into modern/WUS (Wuschel), middle, and old evolutionary branches [19], but those in moso bamboo were only divided into modern branch/WUS and ancient evolutionary branches. Furthermore, there were fewer members of the ancient evolutionary branch than in *Arabidopsis* and rice, suggesting that the bamboo WOX class might have a unique pattern of evolution. 

### 4.2. Gene Structure and Conserved Domains may be Related to the Conserved Function

In this study, 94 of 115 *PeHB*s (85%) had fewer than 10 introns, and only 21 *PeHB*s had more than 10 introns. Half of the members of the PLINC class had no introns, and the remaining genes contained at least one intron. Overall, the number of introns was similar in the same class. For example, the members of the ZIP I class had one or two introns, and those of the ZIP III class contained more introns than the ZIP I, II, and IV classes, but they were relatively conserved, similar to those in grapes [35]. In general, the gain and loss of exons or introns may be the main cause of functional differences, contributing to the functional diversification of these genes [31]. In addition, we identified 20 highly conserved motifs in 115 PeHBs. Different motifs were conserved and their distribution tended to be similar in each class. These results supported the phylogenetic relationship between PeHBs in moso bamboo. Moreover, these motifs were highly conserved during evolution, suggesting that they were possibly correlated to their function.

### 4.3. Diverse Expression of PeHBs in Different Tissues

Based on the expression patterns of *PeHB*s in different tissues and developmental stages in moso bamboo, it was found that they had different constitutive and tissue specific expression patterns. For examples, some *PeHB*s such as the members of the HD-ZIP III, SAWADEE, and NDX classes were highly expressed in all tissues, suggesting that they might be involved in various biological processes and play important roles in the growth and development of moso bamboo. By contrast, *PeHB017* had a very low expression level in all tissues, suggesting that it may be involved in other biological processes. In addition, the expression levels of some *PeHB*s in the PHD and HD-ZIP IV classes in roots were significantly lower than those in other tissues, indicating that they may play a smaller role in the root-related growth and development process. Similarly, most members of the BEL class were highly expressed in the leaves and panicles, but relatively low in the other four tissues, especially in the shoots. On the other hand, *PeHB*s belonging to the PLINC class showed dominant expression in shoots, and were relatively low in the other bamboo tissues. 

### 4.4. The 19 PeHBs Play Important Roles in Bamboo Shoots Undergoing Lignification

Under natural conditions, with the increase in shoot height, the lignification degree in the cell wall of shoots was intensified. The lignification degree of winter bamboo shoots increased in a passive state with the prolongation of storage. All 19 *PeHB*s were upregulated except *PeHB005*, and showed a downregulation in the highest shoots and in the winter shoots at the end of storage. However, the upregulated *PeHB*s demonstrated different expression patterns. For examples, the expression abundance of eight *PeHB*s increased continuously along with the increase of lignification degree in different height shoots and the winter shoots during storage, suggesting that these genes play positive regulatory roles with a continuously rising pattern in the process of lignification. Meanwhile, five upregulated genes (*PeHB019*, *PeHB064*, *PeHB070*, *PeHB088*, and *PeHB100*) showed a similar trend of first rising and then falling with the maximum appearing in 1.0-m or 3.0-m shoots and the winter shoots after 3 d or 6 d storage. *PeHB005* was the only one downregulated in both different height shoots and the winter shoots during storage. These results indicated that the lignification process was a complicated regulatory network associated with multiple *PeHB*s. 

### 4.5. Lignin Synthesis Regulated by a Network

The plant lignification process is a complex process of cell differentiation and development, which is driven by the co-expression of many transcription regulators and functional genes [63,64,65,66,67]. Studies have shown that KNAT7 interacts with the TALE homeodomain protein BLH6, and the KNAT7–BLH6 complex operates as a repression module in secondary cell wall formation [68]. In this study, one member of the KNOX class (*PeHB074*) and two members of the BEL class (*PeHB031*, *PeHB109*) were positively co-expressed in the network, which was supported by the qRT-PCR results of them expressed in the shoots undergoing lignification. In addition, many studies have reported that TFs can regulate the lignin pathway by binding with the key structural genes involved in lignin synthesis [69]. For example, LTF1 acts as a regulator restraining lignin biosynthesis in poplar, which binds the promoter of a key lignin biosynthetic gene encoding 4-coumarate-CoA ligase (4CL) to regulate lignin biosynthesis [66]. In this study, a KNOX binding site was found in the promoter of *Pe4CL*, *PeC3H*, *PeCCR*, and *PeCOMT*, and three *PeHB*s of the KNOX class showed a positive co-expression correlation with these lignin synthesis genes. The co-expression network of *PeHB*s, and PPI of PeHBs, as well as the regulatory roles of PeHBs on structural genes indicated that lignification might be a complicated regulatory network in moso bamboo, which needs further experimental verification.

## 5. Conclusions

Lignification is considered to be a key step in timber formation, and some HB genes are regulators of lignin biosynthesis. The 115 *PeHB*s identified from moso bamboo were classified into 13 classes, which had different expression patterns in various tissues. Nineteen *PeHB*s were predicted to be related to lignin biosynthesis and validated by qRT-PCR, in which 10 *PeHB*s had co-expression correlations, and three members of the KNOX class were hub proteins that interacted with other TFs. PeHB037 interacted with PeHB057 in yeast, which further proved that the PPI prediction was correct. With the increasing degree of lignification, the expression patterns of the 19 *PeHB*s in different height bamboo shoots were similar to those in winter shoots during storage, and three *PeHB*s of the KNOX class had a positive correlation with those of four lignin synthesis genes (*Pe4CL*, *PeC3H*, *PeCCR*, and *PeCOMT*). These results indicated that the involvement of *PeHB*s in bamboo lignification was a multi-level regulatory network.

## Supplementary Materials

The following are available online at https://www.mdpi.com/2218-273X/9/12/862/s1. **Figure S1**: Multiple sequence alignment with the amino acid sequences of HD from different classes in *Phyllostachys edulis* (Pe), *Arabidopsis thaliana* (At), and *Oryza sativa* (Os). Figure S2: Phylogenetic tree based on the amino acid sequences of HB proteins of *Phyllostachys edulis* (Pe), *Arabidopsis thaliana* (At), and *Oryza sativa* (Os). Figure S3: Gene structures of *PeHB*s in moso bamboo. Figure S4: Information of the 20 motifs in PeHBs. Figure S5: GO analysis of *PeHB*s. Figure S6: Transactive analysis of four members belonging to the KNOX class in yeast. The control vectors and fusion constructs of four KNOX genes were transformed into AH109 yeast cells respectively, and inoculated onto SD/−Trp and SD/-Ade /−Leu/−Trp/X-a-GAL plates for further selection. Figure S7: The content changes of cellulose and hemicellulose in winter bamboo shoots during storage. Table S1: Primers used in qRT-PCR. Table S2: Primers used for the construction of gene expression vectors. Table S3: Basic characteristics of the putative proteins encoded by *PeHB*s. Table S4: Classification of HB proteins retrieved from different plants. Table S5: GO enrichment of 113 *PeHB*s. Table S6: GO terms of *PeHB*s. Table S7: The co-expression gene pair in the 19 *PeHB*s. Table S8: The interacted pairs in the PPI network.

## Abbreviations

4CL, 4-coumarate-CoA ligase; BEL, Bel1-like homeodomain; bHLH, basic Helix-Loop-Helix; C3H, coumarate-3-hych’oxylase; CCR, cinnamoyl-CoA reductase; cDNA, complementary deoxyribonucleic acid; COMT, caffeic acid-O-methyltransferase; DDT, DNA binding homeobox and different transcription factors; GO, Gene Ontology; HB, homeobox; HD, homeodomain; HD-ZIP, homeodomain-leucine-zipper; IQD, IQ67-domain; KNOX, knotted-like homeobox; LAC, Laccases; LD, luminidependens homeobox; MYB, myeloblastosis; NCBI, National Center for Biotechnology Information; NDX, nodulin homeobox; PCR, polymerase chain reaction; PHD, plant homeodomain; PINTOX, plant interactor homeobox; PLINC, plant zinc finger; PPI, protein–protein interaction; qRT-PCR, quantitative real time PCR; SBP, Squamosa promoter binding protein; SD, Synthetic Dropout Medium; TALE, three-amino acid loop extension; TCP, teosinte branched 1, cycloidea, and proliferating cell factors; TIGR, the Institute of Genomic Research Database; TF, transcription factor; WOX, Wuschel like homeobox.
