# Supplementary material for: Identification of Homeobox Genes Associated with Lignification and Their Expression Patterns in Bamboo Shoots
Source: Biomolecules. 2019 Dec 11;9(12):862. doi: 10.3390/biom9120862 (PMC6995565; doi:10.3390/biom9120862)

Figure S1: Multiple sequence alignment with the amino acid sequences of HD in different classes of *Phyllostachys edulis* (Pe), *Arabidopsis thaliana* (At), and *Oryza sativa* (Os).

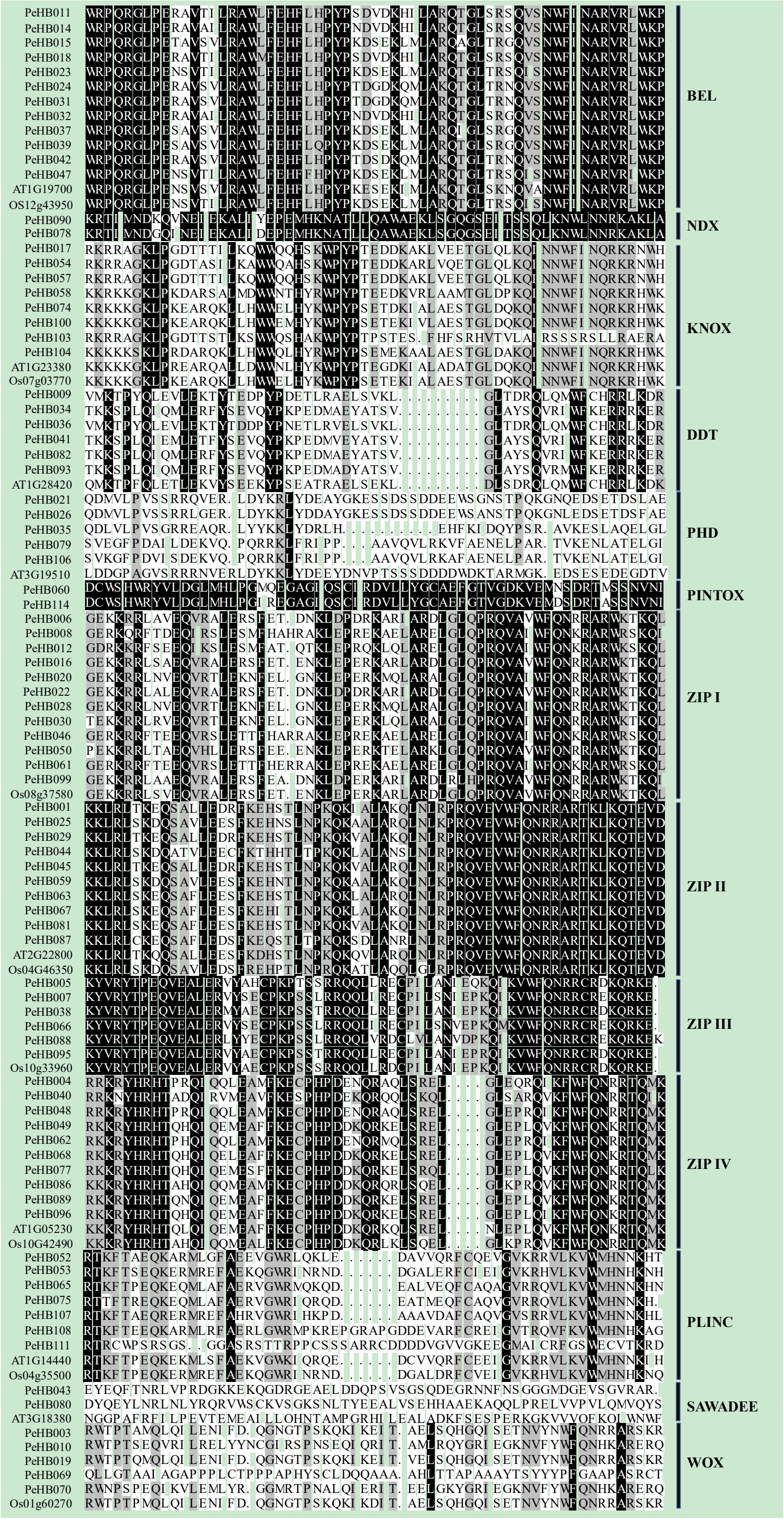

Figure S2: Phylogenetic tree based on the amino acid sequences of HB proteins of *Phyllostachys edulis* (Pe), *Arabidopsis thaliana* (At), and *Oryza sativa* (Os).

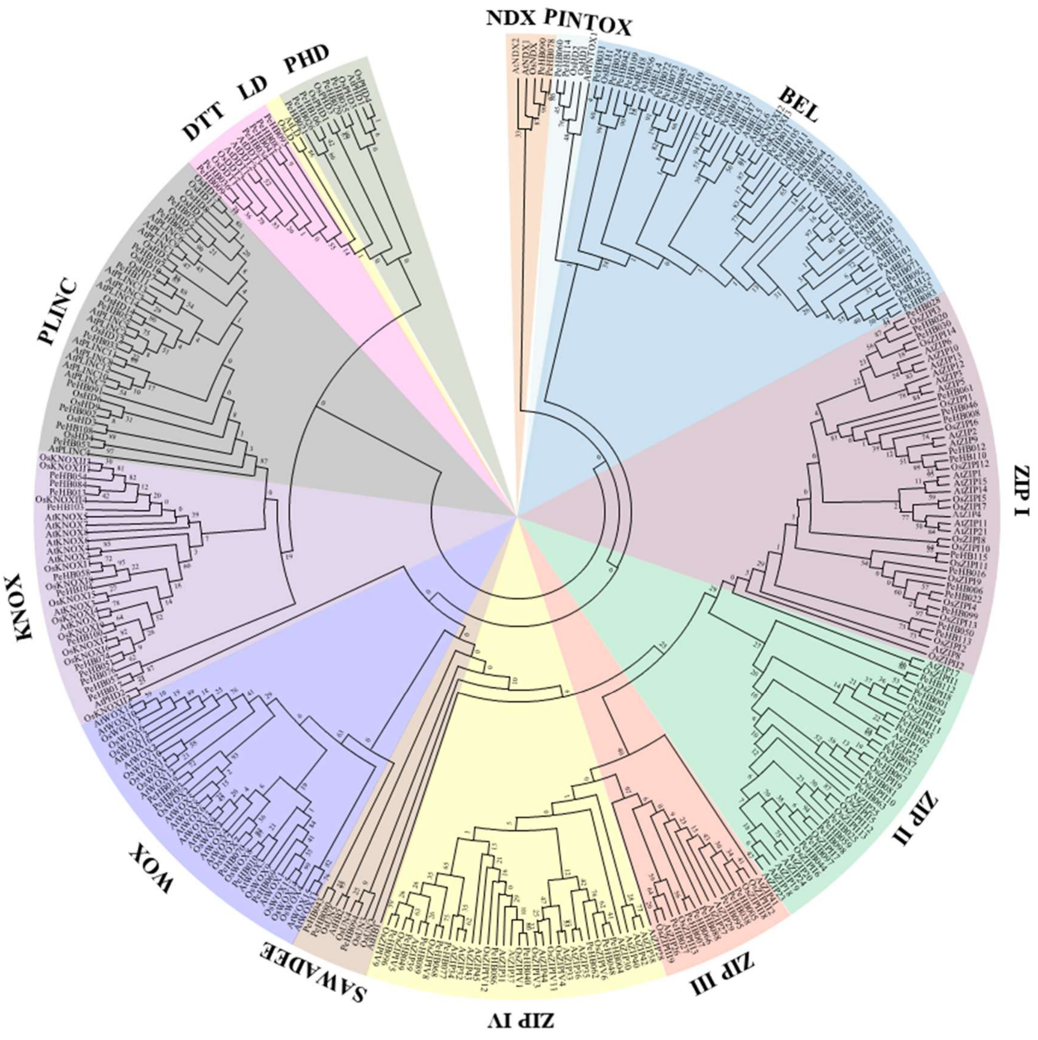

Figure S3: Gene structures of *PeHB*s in moso bamboo.

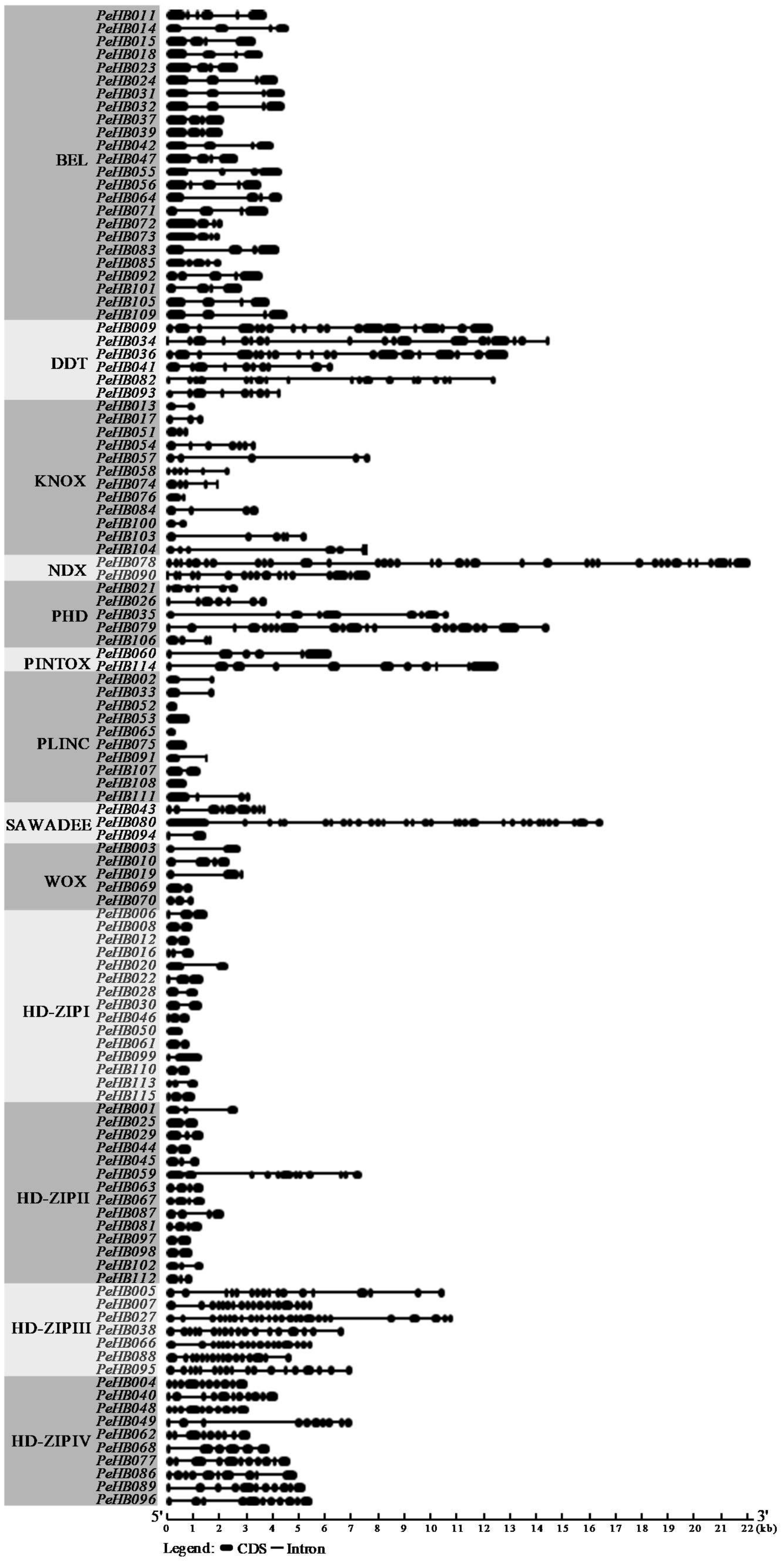

Figure S4: Information of the 20 motifs in PeHBs.

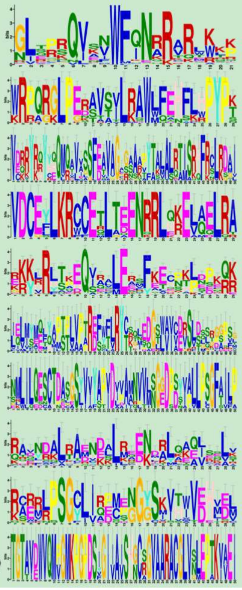

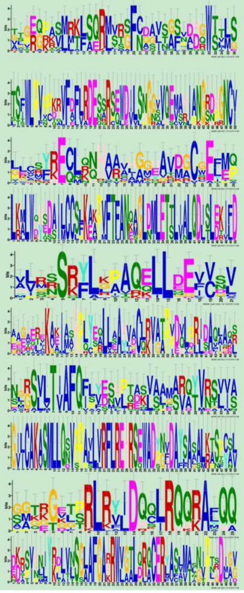

Motif	E-value	Sites	Width	Motif	E-value	Sites	Width
motif 1	3.4e-1062	95	21	motif 11	6.1e-171	15	36
motif 2	6.8e-578	29	29	motif 12	1.8e-169	8	50
motif 3	5.4e-595	21	50	motif 13	9.1e-168	18	29
motif 4	3.3e-569	46	29	motif 14	2.5e-166	7	50
motif 5	1.7e-239	14	29	motif 15	8.1e-152	23	21
motif 6	2.3e-288	15	50	motif 16	3.0e-173	12	47
motif 7	6.3e-224	10	50	motif 17	2.7e-143	13	35
motif 8	5.4e-215	30	29	motif 18	3.4e-153	7	50
motif 9	5.0e-203	16	29	motif 19	1.7e-136	15	25
motif 10	1.4e-201	7	50	motif 20	1.4e-135	8	50

Figure S5: GO analysis of *PeHB*s.

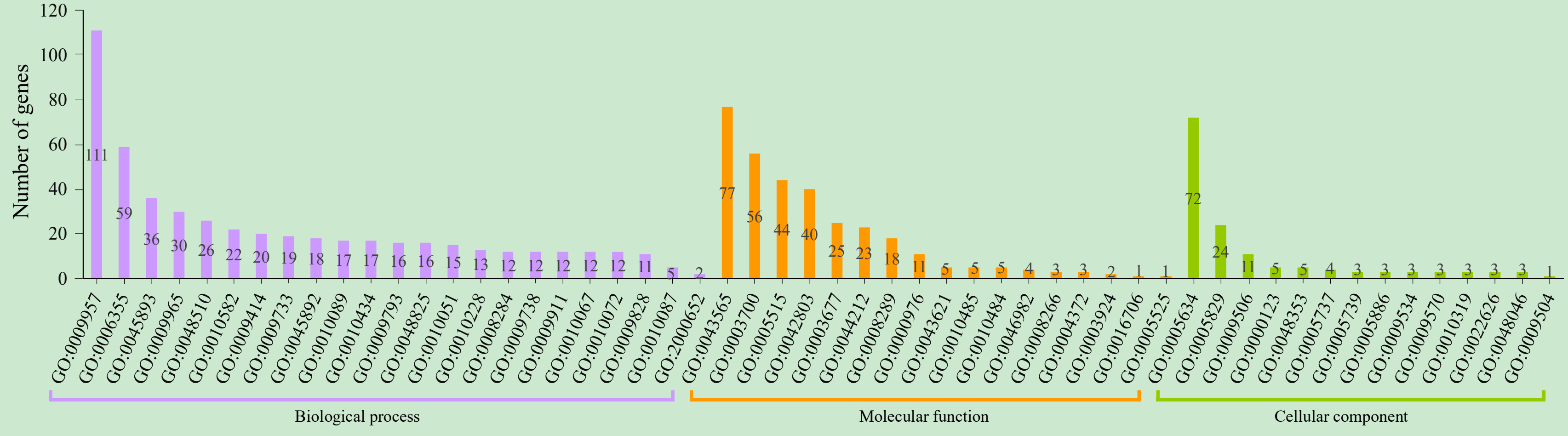

Figure S6: Transactive analysis of four members belonging to KNOX class in yeast. The control vectors and fusion constructs of four KNOX genes were transformed into AH109 yeast cells respectively, and inoculated onto SD/−Trp and SD/-Ade /−Leu/−Trp/X-a-GAL plates for further selection.

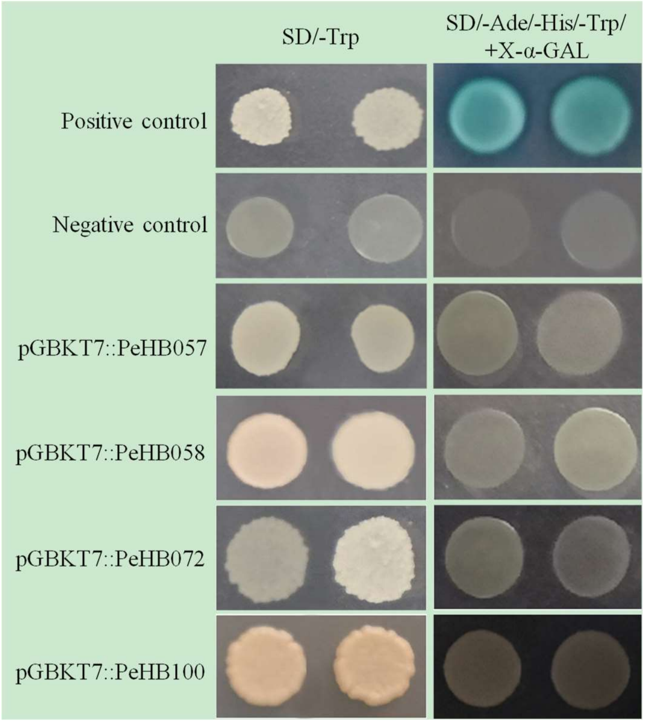

Figure S7: The content changes of cellulose (a) and hemicellulose (b) in winter bamboo shoots during storage. Asterisks indicated a significant difference between the storage shoots and the fresh shoots (*p < 0.05).

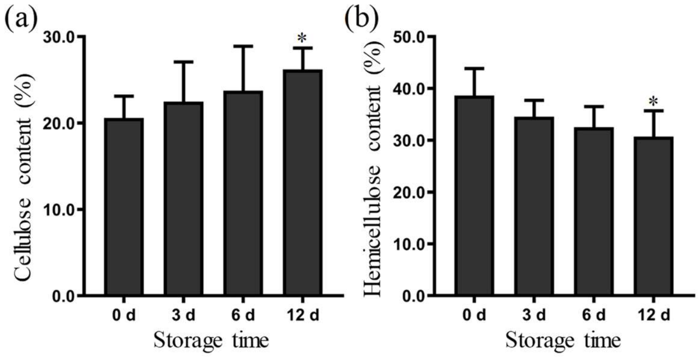

Supplement: Supplementary file 1 [file biomolecules-09-00862-s001.zip › Supplementary Figures.pdf]
