# Supplementary material for: Identification of Homeobox Genes Associated with Lignification and Their Expression Patterns in Bamboo Shoots
Source: Biomolecules. 2019 Dec 11;9(12):862. doi: 10.3390/biom9120862 (PMC6995565; doi:10.3390/biom9120862)
Supplement: Supplementary file 1 [file biomolecules-09-00862-s001.zip › Supplementary Tables.pdf]

Table S1. Primers used in qRT-PCR.

Gene name	Forward primer (5′-3′)	Reverse primer (5′-3′)
PeHB005	TCCACCAGCGGTTCTGGTTCG	ATGGGCCGCAACCCAGGAAG
PeHB007	AGCGGGTGTACAGCGAGTGC	CCGTTGCTTCTCGCGGCATC
PeHB027	TGGCGGTCCATCAGGGCCTA	GAGCCACCTCCCTCGCATGG
PeHB038	CCGGCTGGTGTGTGCTTGTC	CGAAGCAGAAGGCCAGGCAGT
PeHB066	CGATTGCCGGTGCGTGGATG	GGGCCGCCAGTGGATTGAGT
PeHB088	GCAGCTCTACGCTCCGACGA	GCATGCTTGGGCCTCCTTGC
PeHB095	CAGCAGCTTCTGCGCGAGTG	GGAAGCCTCCTTCCGCTGCT
PeHB024	ATTCGCGGCGATGGCTCTGA	GCTTGTGTTGCGCAGCTGGT
PeHB031	CCCGGGACAGGTGAGCTTCG	CCACCGACACCGACCCAGAC
PeHB042	ACATTGCCGAGCCGCTTCCA	CTGGCCTCCGAACGAGCCAA
PeHB064	CTTCCGACGCGTGAGGAGCA	TGGAACTCGGCAGCGACAGG
PeHB109	CAGCTGCGCAACACGAGCAA	CAGCCAAGCGCGAAGAACGG
PeHB019	TGCAGGAATGAGGCTTGGCA	AGCTGCATCTGGGTTGGTGT
PeHB070	GCGGCCTCCTCTTCATCTCC	GTCAGGCGGAGCGATCATCT
PeHB017	GGCTTCGGCCCGCTCCTAAT	TCCCGGCCCTCCTCTTCCTC
PeHB057	GGGCGACGATGTCGGAGGAC	GCTCCTGCCTGACCCTCTCC
PeHB074	TGCTGCACTGGTGGGAGCTG	GGCTTCCAGTGCCGCTTCCT
PeHB100	GCCGCCTACATGGACTGCCA	ATCGGCCGCGTCAGCTCTTC
PeHB104	GTCCTTGCCGGGAGGACGAC	AGTGCAGCTGCCACCAGTGT
Pe4CL	GTGAGGAACGCCGAGCTCAA	CCACCCGTCCTTGTCGATGG
PeCCR	GGTTGTGCTCGGCAAACTGC	GTAGGCGTGCGACTCGTTCA
PeCOMT	CGATGAGGAGGCGTGCATGT	TCCAGCAGGCCCAGCTCTAT
PeC3H	ACCGTGTTGTTGGCCGTGAT	ACTCCTTGACGACGGCTTGC
PeNTB	TCTTGTTTGACACCGAAGAGGAG	AATAGCTGTCCCTGGAGGAGTTT

Table S2. Primers used for the construction of gene expression vectors.

Gene name	Forward primer (5′-3′)	Reverse primer (5′-3′)
PeHB057	CCCATATGATGCAAGGTGGTGATCAAGGAG	CGGGATCCTCACCTTTTACGCTTTGATTTAAGG
PeHB058	CCCATATGATGGAGGATTTGTACAGCATCCACC	CGGAATTCTCATGGTCCGATCGTGCCCG
PeHB072	CCCATATGATGTCATCTTACCCTAACAA	CGGGATCCTCACGAACCGAGGCGGTACA
PeHB100	CCCATATGATGGATCAGAGCTTTGGGAATCTTG	CGGGATCCTCACGAACCGAGGCGGTACAT
PeHB037	CGCCATATGGATGGCCACATTCTTCTCCACC	CGCGGATCCGTCATGCCACAAAATCGTGCA

Table S3. Basic characteristics of the putative proteins encoded by *PeHB*s.

Gene name	BambooGDB assembly name	Peptide length (aa)	Molecular weight (kDa)	pI	Instability index	GRAVY	Subcellular localization
PeHB001	PH01000007G1150	287	30651.49	8.12	54.06	-0.510	Nucleus
PeHB002	PH01000008G0200	179	18617.92	5.61	60.00	-0.280	Nucleus
PeHB003	PH01000009G1450	264	29586.87	5.79	55.48	-0.791	Nucleus
PeHB004	PH01000014G2500	694	77677.36	8.41	58.81	-0.451	Nucleus
PeHB005	PH01000017G2520	795	87179.36	6.25	54.21	-0.158	Nucleus
PeHB006	PH01000038G2120	310	34017.28	4.95	49.10	-0.756	Nucleus
PeHB007	PH01000039G0640	857	93198.92	6.06	47.36	-0.179	Nucleus
PeHB008	PH01000042G1220	255	28373.51	5.11	68.94	-0.725	Nucleus
PeHB009	PH01000046G0140	1830	203663.3	5.36	52.74	-0.754	Nucleus
PeHB010	PH01000057G1290	426	45076.34	8.90	56.27	-0.296	Nucleus
PeHB011	PH01000059G1030	613	66401.33	6.62	45.04	-0.449	Nucleus
PeHB012	PH01000060G1850	232	25701.44	5.51	49.64	-0.860	Nucleus
PeHB013	PH01000061G1320	145	15663.06	6.36	47.85	0.135	Nucleus
PeHB014	PH01000083G0580	447	49018.19	7.01	50.09	-0.476	Nucleus
PeHB015	PH01000103G0430	600	64334.34	5.93	46.28	-0.492	Nucleus
PeHB016	PH01000104G1190	262	28364.32	4.56	52.65	-0.598	Nucleus
PeHB017	PH01000107G0940	300	33735.19	5.77	62.83	-0.550	Nucleus
PeHB018	PH01000107G1130	572	61300.45	6.18	50.52	-0.455	Nucleus
PeHB019	PH01000112G0590	271	30498.88	5.71	53.22	-0.848	Nucleus
PeHB020	PH01000113G0550	324	35537.89	5.95	60.06	-0.672	Nucleus
PeHB021	PH01000114G0530	396	43667.98	4.58	48.91	-0.743	Nucleus
PeHB022	PH01000129G1060	305	33389.62	4.75	49.56	-0.676	Nucleus
PeHB023	PH01000134G0410	648	71880.09	5.73	48.34	-0.700	Nucleus
PeHB024	PH01000139G0610	596	63637.11	7.52	68.37	-0.438	Nucleus
PeHB025	PH01000171G1110	321	34853.47	9.24	64.24	-0.676	Nucleus
PeHB026	PH01000174G0140	426	46712.88	4.64	51.75	-0.595	Nucleus
PeHB027	PH01000183G0940	1287	139540.0	6.03	49.40	-0.182	Nucleus
PeHB028	PH01000188G0440	244	27129.47	6.01	60.24	-0.788	Nucleus
PeHB029	PH01000193G1220	303	31909.85	9.02	52.65	-0.549	Nucleus
PeHB030	PH01000219G0690	280	30409.88	6.23	64.53	-0.728	Nucleus
PeHB031	PH01000223G1160	583	62258.61	7.88	49.41	-0.469	Nucleus
PeHB032	PH01000246G1150	451	49777.32	7.28	47.60	-0.490	Nucleus
PeHB033	PH01000273G0040	182	18543.44	6.09	61.07	-0.564	Nucleus
PeHB034	PH01000279G0700	1297	145139.1	6.26	53.48	-0.517	Nucleus
PeHB035	PH01000284G1150	802	88407.08	5.44	52.45	-0.841	Nucleus
PeHB036	PH01000298G0910	1807	201855.1	5.43	53.80	-0.789	Nucleus
PeHB037	PH01000299G1040	579	63030.88	5.92	51.50	-0.583	Nucleus
PeHB038	PH01000305G0590	850	93516.98	5.71	50.45	-0.102	Nucleus
PeHB039	PH01000312G0600	577	62669.36	6.06	51.40	-0.581	Nucleus

PeHB040	PH01000336G0690	743	81605.92	6.90	51.51	-0.495	Nucleus
PeHB041	PH01000340G1000	668	75955.06	8.85	54.54	-0.374	Nucleus
PeHB042	PH01000348G0740	565	61080.39	7.86	49.73	-0.482	Nucleus
PeHB043	PH01000384G0430	594	68379.56	9.05	40.35	-0.533	Mitochondrial
PeHB044	PH01000407G0480	254	26986.68	9.15	63.23	-0.364	Nucleus
PeHB045	PH01000434G0400	255	28112.94	8.12	58.86	-0.578	Nucleus
PeHB046	PH01000496G0570	206	23202.81	5.26	69.75	-0.792	Nucleus
PeHB047	PH01000496G0760	648	72038.28	5.46	48.78	-0.694	Nucleus
PeHB048	PH01000517G0290	694	76122.62	5.93	46.92	-0.249	Nucleus
PeHB049	PH01000558G0420	552	61568.86	8.35	52.79	-0.412	Nucleus
PeHB050	PH01000569G0270	328	35851.48	4.76	53.86	-0.614	Nucleus
PeHB051	PH01000589G0040	188	20626.46	6.12	47.87	-0.266	Nucleus
PeHB052	PH01000616G0210	271	29362.17	9.41	68.12	-0.683	Nucleus
PeHB053	PH01000630G0150	266	27625.82	8.14	8.14	-0.521	Nucleus
PeHB054	PH01000735G0590	350	39369.86	9.02	49.81	-0.603	Nucleus
PeHB055	PH01000754G0440	611	63857.96	5.53	42.64	-0.344	Nucleus
PeHB056	PH01000759G0070	584	62882.32	8.85	54.25	-0.509	Nucleus
PeHB057	PH01000759G0180	316	34759.26	5.76	58.44	-0.490	Nucleus
PeHB058	PH01000823G0400	296	32503.51	5.50	47.76	-0.606	Nucleus
PeHB059	PH01000841G0210	837	94240.61	8.86	45.22	-0.604	Nucleus
PeHB060	PH01000881G0100	670	74970.64	6.35	49.73	-0.528	Extracellular
PeHB061	PH01000922G0150	228	25311.79	4.80	66.95	-0.759	Nucleus
PeHB062	PH01000928G0560	579	63412.73	6.33	46.44	-0.113	Nucleus
PeHB063	PH01000950G0510	327	34909.15	6.97	71.34	-0.477	Nucleus
PeHB064	PH01000979G0260	473	50691.48	8.96	62.75	-0.306	Nucleus
PeHB065	PH01000983G0460	260	28123.52	9.27	76.38	-0.795	Nucleus
PeHB066	PH01000996G0010	857	93186.01	6.08	48.67	-0.172	Nucleus
PeHB067	PH01001036G0340	335	35530.78	6.96	67.32	-0.462	Nucleus
PeHB068	PH01001046G0030	592	63978.89	5.94	47.25	-0.377	Nucleus
PeHB069	PH01001057G0130	262	27839.52	8.10	71.68	-0.285	Nucleus
PeHB070	PH01001058G0600	217	24273.86	9.25	63.41	-0.501	Nucleus
PeHB071	PH01001092G0270	504	53416.37	6.41	38.75	-0.502	Nucleus
PeHB072	PH01001144G0090	542	59568.28	6.41	57.16	-0.323	Nucleus
PeHB073	PH01001180G0090	528	57633.83	6.35	60.62	-0.323	Nucleus
PeHB074	PH01001206G0320	330	36362.98	5.82	45.38	-0.582	Nucleus
PeHB075	PH01001208G0150	228	24935.95	9.24	83.81	-0.814	Nucleus
PeHB076	PH01001248G0260	171	17048.97	4.58	37.96	0.081	Nucleus
PeHB077	PH01001350G0200	859	93708.15	6.45	48.88	-0.384	Nucleus
PeHB078	PH01001431G0050	2157	241391.0	5.91	46.84	-0.359	Nucleus
PeHB079	PH01001474G0470	1721	190303.1	6.09	55.08	-0.631	Nucleus
PeHB080	PH01001544G0150	1903	214535.2	6.01	49.62	-0.281	Plasma membrane
PeHB081	PH01001550G0050	299	32209.07	9.11	73.87	-0.637	Nucleus
PeHB082	PH01001681G0050	823	91382.44	6.70	63.76	-0.577	Nucleus

PeHB083	PH01001759G0460	585	61337.08	6.17	44.94	-0.432	Nucleus
PeHB084	PH01001802G0120	290	31968.25	6.18	53.29	-0.343	Nucleus
PeHB085	PH01001846G0090	463	50186.73	8.93	58.97	-0.353	Nucleus
PeHB086	PH01001857G0080	769	83565.32	5.48	43.42	-0.270	Nucleus
PeHB087	PH01001885G0240	331	36083.53	6.36	64.57	-0.601	Nucleus
PeHB088	PH01001892G0010	781	85549.96	5.94	54.10	-0.092	Nucleus
PeHB089	PH01001914G0110	785	85343.41	5.35	45.04	-0.293	Nucleus
PeHB090	PH01001950G0130	935	103566.10	5.95	48.03	-0.303	Nucleus
PeHB091	PH01002025G0150	159	17394.12	11.35	79.13	-0.719	Nucleus
PeHB092	PH01002124G0100	618	64813.09	6.33	39.67	-0.377	Nucleus
PeHB093	PH01002136G0270	467	53248.88	9.06	64.82	-0.687	Nucleus
PeHB094	PH01002436G0170	168	18937.52	5.03	48.66	-0.417	Nucleus
PeHB095	PH01002498G0270	858	94225.78	5.59	50.80	-0.098	Nucleus
PeHB096	PH01002523G0060	800	86771.15	5.73	45.5	-0.257	Nucleus
PeHB097	PH01002645G0080	257	27015.87	9.18	63.91	-0.282	Nucleus
PeHB098	PH01002645G0100	271	28685.29	9.37	57.47	-0.439	Nucleus
PeHB099	PH01002691G0230	289	31676.79	5.10	56.81	-0.700	Nucleus
PeHB100	PH01002967G0190	345	38130.06	6.51	44.66	-0.671	Nucleus
PeHB101	PH01003295G0040	458	51217.18	6.05	46.59	-0.697	Nucleus
PeHB102	PH01003417G0160	246	27004.54	8.97	55.74	-0.668	Nucleus
PeHB103	PH01003599G0030	307	33472.53	5.77	54.65	-0.473	Nucleus
PeHB104	PH01003805G0010	354	39623.6	6.09	54.12	-0.597	Nucleus
PeHB105	PH01003816G0150	570	60739.05	6.58	43.62	-0.379	Nucleus
PeHB106	PH01004013G0070	219	24224.6	4.31	53.12	-0.700	Nucleus
PeHB107	PH01004020G0070	324	35767.83	6.53	89.28	-0.938	Nucleus
PeHB108	PH01004023G0120	217	23097.81	8.45	63.41	-0.782	Nucleus
PeHB109	PH01004298G0140	554	59669.85	9.07	50.61	-0.491	Nucleus
PeHB110	PH01004339G0170	231	25658.56	5.27	50.37	-0.808	Nucleus
PeHB111	PH01004504G0130	362	37181.13	7.17	60.89	-0.653	Nucleus
PeHB112	PH01004633G0110	228	24955.78	8.47	65.76	-0.854	Nucleus
PeHB113	PH01004993G0070	325	35365.87	4.64	52.59	-0.558	Nucleus
PeHB114	PH01005072G0040	1137	129019.7	8.24	56.15	-0.763	Nucleus
PeHB115	PH01005345G0020	264	28518.47	4.70	53.46	-0.656	Nucleus

Table S4. Classification of HB proteins retrieved from different plants.

Class	Arabidopsis	Poplar	Rice	Maize	Moso bamboo
HD-ZIP I	15	22	14	24	15
HD-ZIP II	10	16	14	17	14
HD-ZIP III	4	8	4	8	7
HD-ZIP IV	16	15	10	21	10
BEL	13	19	13	17	24
KNOX	8	15	11	13	12
WOX	16	19	19	16	5
LD	1	2	1	1	0
PHD	2	4	2	5	5
PLINC	14	17	11	17	10
DDT	3	7	2	4	6
PINTOX	1	1	2	1	2
NDX	1	2	1	1	2
SAWADEE	2	1	2	3	3
Total	106	148	106	148	115

Table S5. GO enrichment of 113 *PeHB*s

GO-term	Log_2-Enrichment	p-value	Description
GO:0000741	4.08	2.68E-04	Karyogamy
GO:0001944	5.24	5.74E-07	Vasculature development
GO:0003002	3.71	2.72E-17	Regionalization
GO:0006349	5.89	2.37E-15	Regulation of gene expression by genetic imprinting
GO:0006355	3.16	5.16E-75	Regulation of transcription, DNA-templated
GO:0006473	4.1	3.16E-05	Protein acetylation
GO:0006475	4.1	3.16E-05	Internal protein amino acid acetylation
GO:0006544	4.51	8.97E-04	Glycine metabolic process
GO:0006563	3.69	0.004635898	L-serine metabolic process
GO:0008283	4.37	2.16E-20	Cell proliferation
GO:0008284	6.01	9.13E-19	Positive regulation of cell proliferation
GO:0009414	2.58	3.32E-10	Response to water deprivation
GO:0009416	2.71	6.75E-21	Response to light stimulus
GO:0009610	4.78	3.88E-05	Response to symbiotic fungus
GO:0009615	2.46	0.005791109	Response to virus
GO:0009626	2.56	0.038662376	Plant-type hypersensitive response
GO:0009637	3.61	1.61E-07	Response to blue light
GO:0009641	4.46	9.34E-06	Shade avoidance
GO:0009691	6.36	1.51E-05	Cytokinin biosynthetic process
GO:0009722	7.36	1.10E-06	Detection of cytokinin stimulus
GO:0009725	2.19	8.42E-23	Response to hormone
GO:0009727	6.28	2.75E-10	Detection of ethylene stimulus
GO:0009733	3.01	9.82E-14	Response to auxin
GO:0009735	2.69	9.36E-09	Response to cytokinin
GO:0009736	3.46	0.001381705	Cytokinin-activated signaling pathway
GO:0009738	3.66	1.22E-14	Abscisic acid-activated signaling pathway
GO:0009788	4.21	3.02E-07	Negative regulation of abscisic acid-activated signaling pathway
GO:0009793	3.49	4.88E-30	Embryo development ending in seed dormancy
GO:0009828	6.71	2.57E-18	Plant-type cell wall loosening
GO:0009838	3.75	0.004138073	Abscission
GO:0009845	2.87	3.26E-06	Seed germination
GO:0009853	3.41	0.008016883	Photorespiration
GO:0009880	4.89	2.25E-14	Embryonic pattern specification
GO:0009908	3.89	9.27E-56	Flower development
GO:0009911	4.59	3.50E-11	Positive regulation of flower development
GO:0009942	5.46	2.61E-07	Longitudinal axis specification
GO:0009943	5.43	1.86E-16	Adaxial/abaxial axis specification
GO:0009947	7.78	5.36E-12	Centrolateral axis specification

GO:0009953	7.78	1.01E-16	Dorsal/ventral pattern formation
GO:0009956	5.09	3.88E-09	Radial pattern formation
GO:0009957	6.85	6.78E-19	Epidermal cell fate specification
GO:0009965	4.64	5.04E-32	Leaf morphogenesis
GO:0009996	7.52	4.99E-22	Negative regulation of cell fate specification
GO:0010016	4.53	3.04E-42	Shoot system morphogenesis
GO:0010017	3.47	2.54E-04	Red or far-red light signaling pathway
GO:0010022	6.15	5.81E-32	Meristem determinacy
GO:0010051	4.06	4.78E-14	Xylem and phloem pattern formation
GO:0010062	7.52	4.99E-22	Negative regulation of trichoblast fate specification
GO:0010067	6.9	8.08E-23	Procambium histogenesis
GO:0010072	6.15	2.18E-19	Primary shoot apical meristem specification
GO:0010073	5.34	2.45E-59	Meristem maintenance
GO:0010076	6.19	1.54E-08	Maintenance of floral meristem identity
GO:0010077	6.19	1.54E-08	Maintenance of inflorescence meristem identity
GO:0010087	5.07	3.28E-27	Phloem or xylem histogenesis
GO:0010089	5.6	5.11E-24	Xylem development
GO:0010091	4.8	8.24E-12	Trichome branching
GO:0010154	3.13	1.86E-33	Fruit development
GO:0010197	4.08	2.68E-04	Polar nucleus fusion
GO:0010201	6.78	8.40E-08	Response to continuous far red light stimulus by the high-irradiance response system
GO:0010218	3.75	7.00E-08	Response to far red light
GO:0010223	4.67	4.37E-06	Secondary shoot formation
GO:0010228	3.3	8.82E-13	Vegetative to reproductive phase transition of meristem
GO:0010321	7.1	2.96E-10	Regulation of vegetative phase change
GO:0010346	4.67	4.37E-06	Shoot axis formation
GO:0010371	6.1	2.23E-08	Regulation of gibberellin biosynthetic process
GO:0010433	7.44	3.25E-32	Bract morphogenesis
GO:0010434	7.44	3.25E-32	Bract formation
GO:0010582	6.15	5.81E-32	Floral meristem determinacy
GO:0010629	2.94	9.83E-16	Negative regulation of gene expression
GO:0010654	7.78	5.36E-12	Apical cell fate commitment
GO:0010865	7.78	5.36E-12	Stipule development
GO:0016049	2.45	4.54E-09	Cell growth
GO:0016573	4.1	3.16E-05	Histone acetylation
GO:0018393	4.1	3.16E-05	Internal peptidyl-lysine acetylation
GO:0018394	4.1	3.16E-05	Peptidyl-lysine acetylation
GO:0019538	-2.06	2.36E-05	Protein metabolic process

GO:0019827	4.96	8.59E-16	Stem cell population maintenance
GO:0021700	2.75	6.16E-07	Developmental maturation
GO:0030308	5.06	4.56E-09	Negative regulation of cell growth
GO:0031047	3.55	4.32E-08	Gene silencing by RNA
GO:0032870	2.67	1.45E-16	Cellular response to hormone stimulus
GO:0034097	7.36	1.10E-06	Response to cytokine
GO:0040007	2.42	3.84E-12	Growth
GO:0040008	3.4	6.98E-12	Regulation of growth
GO:0042335	4.61	3.12E-11	Cuticle development
GO:0043478	6.71	2.57E-18	Pigment accumulation in response to UV light
GO:0043479	6.71	2.57E-18	Pigment accumulation in tissues in response to UV light
GO:0043480	6.71	2.57E-18	Pigment accumulation in tissues
GO:0043481	6.71	2.57E-18	Anthocyanin accumulation in tissues in response to UV light
GO:0045165	5.32	9.38E-24	Cell fate commitment
GO:0045595	5.3	2.49E-22	Regulation of cell differentiation
GO:0045597	6.68	6.26E-13	Positive regulation of cell differentiation
GO:0045893	3.88	1.66E-24	Positive regulation of transcription, DNA-templated
GO:0048262	7.78	1.01E-16	Determination of dorsal/ventral asymmetry
GO:0048263	7.78	1.01E-16	Determination of dorsal identity
GO:0048314	7.29	1.12E-10	Embryo sac morphogenesis
GO:0048437	4.17	7.08E-34	Floral organ development
GO:0048440	5.23	2.50E-28	Carpel development
GO:0048445	6.43	1.40E-15	Carpel morphogenesis
GO:0048446	5.58	1.67E-07	Petal morphogenesis
GO:0048457	7.52	3.20E-11	Floral whorl morphogenesis
GO:0048467	5.08	1.48E-33	Gynoecium development
GO:0048469	3.1	7.88E-07	Cell maturation
GO:0048481	5.29	1.38E-23	Plant ovule development
GO:0048482	7.29	1.12E-10	Plant ovule morphogenesis
GO:0048497	6.78	1.35E-18	Maintenance of floral organ identity
GO:0048506	5.86	1.92E-34	Regulation of timing of meristematic phase transition
GO:0048507	4.66	9.17E-60	Meristem development
GO:0048509	4.99	3.10E-40	Regulation of meristem development
GO:0048518	3.04	5.44E-33	Positive regulation of biological process
GO:0048522	3.23	5.79E-28	Positive regulation of cellular process
GO:0048825	4.92	1.96E-21	Cotyledon development
GO:0048827	3.42	5.90E-36	Phyllome development
GO:0048859	6.14	4.33E-13	Formation of anatomical boundary

GO:0051094	4.15	5.98E-21	Positive regulation of developmental process
GO:0051240	3.73	1.47E-12	Positive regulation of multicellular organismal process
GO:0051301	1.94	0.011816131	Cell division
GO:0055114	-2.93	0.009136956	Oxidation-reduction process
GO:0071345	7.36	1.10E-06	Cellular response to cytokine stimulus
GO:0071369	3.14	1.79E-04	Cellular response to ethylene stimulus
GO:0071514	5.89	2.37E-15	Genetic imprinting
GO:0072358	5.24	5.74E-07	Cardiovascular system development
GO:0072359	5.24	5.74E-07	Circulatory system development
GO:0080006	7.52	3.20E-11	Internode patterning
GO:0080022	3.34	0.009136198	Primary root development
GO:0080060	6.68	6.26E-13	Integument development
GO:0080127	7.78	5.36E-12	Fruit septum development
GO:0080166	7.78	5.36E-12	Stomium development
GO:0090451	7.52	3.20E-11	Cotyledon boundary formation
GO:0090470	7.36	1.10E-06	Shoot organ boundary specification
GO:0090691	6.14	4.33E-13	Formation of plant organ boundary
GO:0090700	6.78	1.35E-18	Maintenance of plant organ identity
GO:0097353	7.78	5.36E-12	Centrolateral pattern formation
GO:0098727	4.96	8.59E-16	Maintenance of cell number
GO:1901420	4.21	3.02E-07	Negative regulation of response to alcohol
GO:1902459	6.93	6.64E-10	Positive regulation of stem cell population maintenance
GO:1903506	3.16	5.16E-75	Regulation of nucleic acid-templated transcription
GO:1903889	7.52	4.99E-22	Negative regulation of plant epidermal cell differentiation
GO:1905392	3.54	2.10E-38	Plant organ morphogenesis
GO:1905422	7.52	4.99E-22	Negative regulation of plant organ morphogenesis
GO:1905958	4.21	3.02E-07	Negative regulation of cellular response to alcohol
GO:0003677	3.36	4.24E-113	DNA binding
GO:0042803	4.72	1.67E-44	Protein homodimerization activity
GO:0016787	-4.02	1.30E-06	Hydrolase activity
GO:0036094	-4.34	1.44E-08	Small molecule binding
GO:0043621	3.14	7.44E-04	Protein self-association
GO:0016491	-2.95	0.008144437	Oxidoreductase activity
GO:0097367	-4.18	1.65E-07	Carbohydrate derivative binding
GO:0004372	5.9	4.39E-05	Glycine hydroxymethyltransferase activity
GO:1901265	-4.27	4.44E-08	Nucleoside phosphate binding
GO:0046982	2.27	0.024604286	Protein heterodimerization activity

GO:0044212	3.94	1.27E-29	Transcription regulatory region DNA binding
GO:0008266	4.41	0.001093749	Poly(U) RNA binding
GO:0043565	4.31	1.44E-99	Sequence-specific DNA binding
GO:0008187	4.41	0.001093749	Poly-pyrimidine tract binding
GO:0010484	7.52	3.20E-11	H3 histone acetyltransferase activity
GO:0008289	3.92	3.81E-15	Lipid binding
GO:0003700	3.57	2.62E-45	Transcription factor activity, sequence-specific DNA binding
GO:0003824	-2.14	2.40E-14	Catalytic activity
GO:0010485	5.78	7.79E-08	H4 histone acetyltransferase activity
GO:0043167	-4.89	1.18E-13	Ion binding
GO:0005634	1.93	5.85E-34	Nucleus
GO:0048353	7.78	5.36E-12	Primary endosperm nucleus
GO:0016020	-3.39	3.96E-12	Membrane
GO:0009536	-2.36	1.67E-04	Plastid
GO:0071944	-2.63	7.71E-06	Cell periphery
GO:0005829	0.94	9.66E-04	Cytosol
GO:0000123	4.58	6.15E-06	Histone acetyltransferase complex

Table S6. GO terms of *PeHB*s.

GO Classification	GO number	Amount	Annotation
Biological process (1080)	GO:0000724	1	Double-strand break repair via homologous recombination
	GO:0000911	2	Cytokinesis by cell plate formation
	GO:0001558	3	Regulation of cell growth
	GO:0001708	3	Cell fate specification
	GO:0001944	5	Vasculature development
	GO:0003677	1	DNA binding
	GO:0006275	1	Regulation of DNA replication
	GO:0006334	1	Nucleosome assembly
	GO:0006349	11	Regulation of gene expression by genetic imprinting
	GO:0006355	59	Regulation of transcription, DNA-templated
	GO:0006544	3	Glycine metabolic process
	GO:0006563	3	L-serine metabolic process
	GO:0007165	5	Signal transduction
	GO:0007623	3	Circadian rhythm
	GO:0008283	6	Cell proliferation
	GO:0008284	12	Positive regulation of cell proliferation
	GO:0008285	4	Negative regulation of cell proliferation
	GO:0009294	1	DNA mediated transformation
	GO:0009409	3	Response to cold
	GO:0009414	20	Response to water deprivation
	GO:0009416	6	Response to light stimulus
	GO:0009555	2	Pollen development
	GO:0009610	4	Response to symbiotic fungus
	GO:0009615	6	Response to virus
	GO:0009626	3	Plant-type hypersensitive response
	GO:0009637	10	Response to blue light
	GO:0009640	4	Photomorphogenesis
	GO:0009641	5	Shade avoidance
	GO:0009646	2	Response to absence of light
	GO:0009651	9	Response to salt stress
	GO:0009691	3	Cytokinin biosynthetic process
	GO:0009722	3	Detection of cytokinin stimulus
	GO:0009723	4	Response to ethylene
	GO:0009727	7	Detection of ethylene stimulus
	GO:0009733	19	Response to auxin
	GO:0009734	5	Auxin-activated signaling pathway
	GO:0009735	9	Response to cytokinin
	GO:0009736	4	Cytokinin-activated signaling pathway
	GO:0009737	6	Response to abscisic acid
	GO:0009738	12	Abscisic acid-activated signaling pathway
	GO:0009739	5	Response to gibberellin
	GO:0009744	3	Response to sucrose

GO:0009788	7	Negative regulation of abscisic acid-activated signaling pathway
GO:0009793	16	Embryo development ending in seed dormancy
GO:0009826	5	Unidimensional cell growth
GO:0009828	11	Plant-type cell wall loosening
GO:0009845	11	Seed germination
GO:0009853	3	Photorespiration
GO:0009880	7	Embryonic pattern specification
GO:0009901	1	Anther dehiscence
GO:0009911	12	Positive regulation of flower development
GO:0009934	4	Regulation of meristem structural organization
GO:0009942	5	Longitudinal axis specification
GO:0009943	5	Adaxial/abaxial axis specification
GO:0009944	7	Polarity specification of adaxial/abaxial axis
GO:0009947	5	Centrolateral axis specification
GO:0009956	7	Radial pattern formation
GO:0009957	111	Epidermal cell fate specification
GO:0009965	30	Leaf morphogenesis
GO:0010015	5	Root morphogenesis
GO:0010016	5	Shoot system morphogenesis
GO:0010017	5	Red or far-red light signaling pathway
GO:0010019	3	Chloroplast-nucleus signaling pathway
GO:0010026	1	Trichome differentiation
GO:0010051	15	Xylem and phloem pattern formation
GO:0010062	11	Negative regulation of trichoblast fate specification
GO:0010067	12	Procambium histogenesis
GO:0010072	12	Primary shoot apical meristem specification
GO:0010073	8	Meristem maintenance
GO:0010075	7	Regulation of meristem growth
GO:0010076	5	Maintenance of floral meristem identity
GO:0010077	5	Maintenance of inflorescence meristem identity
GO:0010078	5	Maintenance of root meristem identity
GO:0010087	5	Phloem or xylem histogenesis
GO:0010089	17	Xylem development
GO:0010091	11	Trichome branching
GO:0010094	4	Specification of carpel identity
GO:0010154	5	Fruit development
GO:0010192	2	Mucilage biosynthetic process
GO:0010197	4	Polar nucleus fusion
GO:0010201	4	Response to continuous far red light stimulus by the high-irradiance response system
GO:0010218	5	Response to far red light
GO:0010223	5	Secondary shoot formation
GO:0010227	4	Floral organ abscission

	GO:0010228	13	Vegetative to reproductive phase transition of meristem
	GO:0010229	1	Inflorescence development
	GO:0010321	5	Regulation of vegetative phase change
	GO:0010371	6	Regulation of gibberellin biosynthetic process
	GO:0010431	2	Seed maturation
	GO:0010434	17	Bract formation
	GO:0010492	5	Maintenance of shoot apical meristem identity
	GO:0010582	22	Floral meristem determinacy
	GO:0010654	5	Apical cell fate commitment
	GO:0010865	5	Stipule development
	GO:0016049	5	Cell growth
	GO:0016573	5	Histone acetylation
	GO:0019760	2	Glucosinolate metabolic process
	GO:0019827	3	Stem cell population maintenance
	GO:0030308	7	Negative regulation of cell growth
	GO:0031047	11	Gene silencing by RNA
	GO:0031507	1	Heterochromatin assembly
	GO:0042335	11	Cuticle development
	GO:0043481	11	Anthocyanin accumulation in tissues in response to UV light
	GO:0045597	7	Positive regulation of cell differentiation
	GO:0045787	1	Positive regulation of cell cycle
	GO:0045892	18	Negative regulation of transcription, DNA-templated
	GO:0045893	36	Positive regulation of transcription, DNA-templated
	GO:0046686	3	Response to cadmium ion
	GO:0048263	7	Determination of dorsal identity
	GO:0048314	5	Embryo sac morphogenesis
	GO:0048364	7	Root development
	GO:0048366	1	Leaf development
	GO:0048446	5	Petal morphogenesis
	GO:0048457	5	Floral whorl morphogenesis
	GO:0048467	5	Gynoecium development
	GO:0048481	6	Plant ovule development
	GO:0048482	5	Plant ovule morphogenesis
	GO:0048497	11	Maintenance of floral organ identity
	GO:0048510	26	Regulation of timing of transition from vegetative to reproductive phase
	GO:0048573	7	Photoperiodism, flowering
	GO:0048574	1	Long-day photoperiodism, flowering
	GO:0048765	11	Root hair cell differentiation
	GO:0048825	16	Cotyledon development
	GO:0048826	3	Cotyledon morphogenesis
	GO:0051301	5	Cell division
	GO:0051455	1	Attachment of spindle microtubules to kinetochore involved in homologous chromosome segregation

	GO:0051726	1	Regulation of cell cycle
	GO:0051754	1	Meiotic sister chromatid cohesion, centromeric
	GO:0055114	2	Oxidation-reduction process
	GO:0070734	1	Histone H3-K27 methylation
	GO:0071345	3	Cellular response to cytokine stimulus
	GO:0071369	7	Cellular response to ethylene stimulus
	GO:0080006	5	Internode patterning
	GO:0080022	3	Primary root development
	GO:0080060	7	Integument development
	GO:0080127	5	Fruit septum development
	GO:0080166	5	Stomium development
	GO:0080167	5	Response to karrikin
	GO:0090451	5	Cotyledon boundary formation
	GO:0090470	4	Shoot organ boundary specification
	GO:1902459	5	Positive regulation of stem cell population maintenance
	GO:2000652	2	Regulation of secondary cell wall biogenesis
Cellular component (143)	GO:0005634	72	Nucleus
	GO:0005829	24	Cytosol
	GO:0009506	11	Plasmodesma
	GO:0000123	5	Histone acetyltransferase complex
	GO:0048353	5	Primary endosperm nucleus
	GO:0005737	4	Cytoplasm
	GO:0005739	3	Mitochondrion
	GO:0005886	3	Plasma membrane
	GO:0009534	3	Chloroplast thylakoid
	GO:0009570	3	Chloroplast stroma
	GO:0010319	3	Stromule
	GO:0022626	3	Cytosolic ribosome
	GO:0048046	3	Apoplast
	GO:0009504	1	Cell plate
Molecular function (323)	GO:0043565	77	Sequence-specific DNA binding
	GO:0003700	56	Transcription factor activity, sequence-specific DNA binding
	GO:0005515	44	Protein binding
	GO:0042803	40	Protein homodimerization activity
	GO:0003677	25	DNA binding
	GO:0044212	23	Transcription regulatory region DNA binding
	GO:0008289	18	Lipid binding
	GO:0000976	11	Transcription regulatory region sequence-specific DNA binding
	GO:0043621	5	Protein self-association
	GO:0010485	5	H4 histone acetyltransferase activity
	GO:0010484	5	H3 histone acetyltransferase activity
	GO:0046982	4	Protein heterodimerization activity
	GO:0008266	3	Poly(U) RNA binding

	GO:0004372	3	Glycine hydroxymethyltransferase activity
	GO:0003924	2	Gtpase activity
	GO:0016706	1	Oxidoreductase activity, acting on paired donors, with incorporation or reduction of molecular oxygen, 2-oxoglutarate as one donor, and incorporation of one atom each of oxygen into both donors
	GO:0005525	1	GTP binding

Table S7. The co-expression gene pair in the 19 *PeHB*s.

Pair number	Gene name	Gene name	Type
1	*PeHB005* (PH01000017G2520)	*PeHB095* (PH01002498G0270)	Negative
2	*PeHB017* (PH01000183G0940)	*PeHB007* (PH01000039G0640)	Positive
3	*PeHB027* (PH01000107G0940)	*PeHB057* (PH01000759G0180)	Positive
4	*PeHB031* (PH01000223G1160)	*PeHB109* (PH01004298G0140)	Positive
5	*PeHB042* (PH01000348G0740)	*PeHB109* (PH01004298G0140)	Positive
6	*PeHB074* (PH01001206G0320)	*PeHB109* (PH01004298G0140)	Positive
7	*PeHB074* (PH01001206G0320)	*PeHB031* (PH01000223G1160)	Positive

Table S8. The interacted pairs in the PPI network.

Pair number	Protein name	Protein name
1	PeHB005 (PH01000017G2520)	PeMYB1 (PH01001558G0360)
2	PeHB005 (PH01000017G2520)	PeMYB16 (PH01000501G0490)
3	PeHB023 (PH01000134G0410)	PeHB057 (PH01000759G0180)
4	PeHB023 (PH01000134G0410)	PeHB058 (PH01000823G0400)
5	PeHB023 (PH01000134G0410)	PeHB100 (PH01002967G0190)
6	PeHB037 (PH01000299G1040)	PeHB057 (PH01000759G0180)
7	PeHB037 (PH01000299G1040)	PeHB058 (PH01000823G0400)
8	PeHB037 (PH01000299G1040)	PeHB100 (PH01002967G0190)
9	PeHB042 (PH01000348G0740)	PeHB054 (PH01000735G0590)
10	PeHB042 (PH01000348G0740)	PeHB100 (PH01002967G0190)
11	PeHB042 (PH01000348G0740)	PebHLH1 (PH01000323G0020)
12	PeHB057 (PH01000759G0180)	PeHB072 (PH01001144G0090)
13	PeHB057 (PH01000759G0180)	PeHB100 (PH01002967G0190)
14	PeHB057 (PH01000759G0180)	PebHLH3 (PH01001119G0330)
15	PeHB057 (PH01000759G0180)	PeMYB7 (PH01001022G0490)
16	PeHB057 (PH01000759G0180)	PeOVATE1 (PH01003968G0140)
17	PeHB058 (PH01000823G0400)	PeHB057 (PH01000759G0180)
18	PeHB058 (PH01000823G0400)	PeHB072 (PH01001144G0090)
19	PeHB058 (PH01000823G0400)	PeHB064 (PH01000979G0260)
20	PeHB064 (PH01000979G0260)	PeHB100 (PH01002967G0190)
21	PeHB100 (PH01002967G0190)	PeHB072 (PH01001144G0090)
22	PeHB100 (PH01002967G0190)	PeOVATE1 (PH01003968G0140)
23	PebHLH1 (PH01000323G0020)	PeHB042 (PH01000348G0740)
24	PebHLH2 (PH01000448G0580)	PeHB057 (PH01000759G0180)
25	PeTCP1 (PH01000005G0450)	PeHB100 (PH01002967G0190)
26	PeOVATE12 (PH01000081G0900)	PeHB100 (PH01002967G0190)
27	PeOVATE12 (PH01000081G0900)	PeHB058 (PH01000823G0400)
